# A study in University of Ruhuna for investigating prevalence, risk factors and remedies for psychiatric illnesses among students

**DOI:** 10.1038/s41598-022-16838-4

**Published:** 2022-07-27

**Authors:** Patikiri Arachchige Don Shehan Nilmantha Wijesekara

**Affiliations:** Department of Electrical and Information Engineering, Faculty of Engineering, University of Ruhuna, Galle, Southern Province 80000 Sri Lanka

**Keywords:** Psychology, Psychiatric disorders, Risk factors, Signs and symptoms, Statistics, Population screening

## Abstract

There is no comprehensive study on the mental health of Sri Lankan undergraduate in higher education, as most existing studies have been done for medical students only. It is unknown how academic and environmental factors contribute for the prevalence of psychiatric illnesses. Further, there is no sufficient information on the student/university based remedies to reduce the psychological distress of students. This research is carried out to find the overall psychological distress, well-being, prevalence percentages of psychiatric illnesses, associated risk factors, and student/university remedies to overcome them. We use standard questionnaires to screen for psychiatric illnesses, and we analyze the responses for our own questionnaire using Binary logistic regression analysis to identify demographic factors, academic factors, and environmental factors causing each mental disorder. We use Pearson correlation coefficient to identify correlation between prevalence of each psychiatric illnesses. All 13 psychiatric illnesses were found with a moderate correlation among diseases having a mean prevalence percentage of 28 and a standard deviation of 14.36, despite the prevalence of well-being factors among students and only 8% are clinically diagnosed. 89% of the students were suffering from at least one psychiatric illness and 68% were found to be psychologically distressed. Sets of overall and individual demographic, academic, and environmental risk factors contributing for the prevalence of a psychiatric illness in general and in particular were identified respectively after a binary logistic regression analysis. 61% of the students don’t receive psychiatric help from the university and are using their own remedies. The universities must consider the environmental and academic risk factors associated with psychiatric illnesses and design curriculum, expand resources, and provide counseling services to reduce the impact of risk factors.

## Introduction

Generalized Anxiety Disorder (GAD) is having fear/worry or distressing thoughts about everyday life that interfere with daily living. A survey done on an online doctoral program has shown that students had anxiety in computer use, internet, and online learning^1^. The review article^2^  discusses different interventions taken by students and faculties to reduce anxiety levels of nursing students. A recent study on college students have proved that highest level of anxiety is caused due to academic distress^3^. According to a study on Chinese overseas students; they have developed high levels of anxiety in education due to the Coronavirus Disease of 2019 (COVID-19) pandemic^4^. It has been found that active learning can either increase or decrease anxiety according to the way it is implemented^5^. It has also been found that the health anxiety which is the fear of getting an illness has been increased over the last decades among students^6^. According to a recent study, the test anxiety of students has been worsened due to online proctoring of examinations^7^. It has been found that female students tend to have higher anxiety levels than male students^8^. Two stage testing has reduced student anxiety compared to traditional one stage tests for students taking introduction to chemistry exams^9^. Mindfulness meditation has proved to reduce the anxiety levels of college students^10^. Animal assisted therapies have been effective in treating anxiety of Nursing students^11^. Another effective solution to reducing student anxiety levels is by biofeedback which is using electronic sensors to know about one’s own body functions and then voluntarily reduce adverse conditions^12^.

There is no evidence to prove whether agoraphobia is existed among students or not^13^. Agoraphobia is an anxiety disorder of fear of too far from being a safe person or place, or avoiding of places or situations which might cause the person to panic or feel trapped.

Only a few evidence exists for the presence of panic disorder among the students also^14^. Panic disorder is feeling of a sudden/unreasonable fear.

Social Anxiety Disorder (SAD)/Social phobia is a fear of being watched or judged by others. It has been found in a group of health science students in Ethiopia and authors claim that it is highly prevalent among the university students^15^. SAD has been found more frequently in female medical students more than other students according to findings of a study conducted among Egyptian university students^16^. SAD has been found to be associated with internet addiction and depression^17^. Some studies show that social phobia can negatively affect for the academic performance^18^.

Next major mental illness is depression which is the mental state of having low mood, reduced energy, and loss of interest. A study in 2013 had showed that one third of university students have depression in average^19^. A similar study shows that medical students have a higher chance of depression than other students^20^. Another study shows that around 10% of medical students have depression to the level of suicidal ideation^21^. Further, there is evidence to prove that Ph.D. students have higher levels of depression^22^. When considering the gender differences among male and female students, male students have been reported to have higher chance of getting depressed^8^. The correlates of depression have been found to be as high academic performance, pressure to succeed, and postgraduate plans^23^. Academic delays due to COVID-19 has also been found to worsen the effects of depression^24^. As depression prevention programs; psycho-education, relaxation techniques, and cognitive monitoring have been effective^25^. Some have suggested Digital mental health intervention programs such as internet based cognitive behavioral therapy to reduce effects of depression^26^. Work in^27^  suggests that social support from family and friends can be used to reduce the effects of depression. According to a study conducted among Sri Lankan undergraduates, 10% of students have shown major depression symptoms^28^. Another similar survey has found that peer students support depressed Sri Lankan students and only a few seeks professional support^29^.

Bipolar Affective Disorder (BAD) is a mood disorder which a person experiences episodes of mania (high mood, high energy, and high interest) and depression interchangeably from time to time. A recent study on bipolar affective disorder shows that even though its prevalence is relatively lower than anxiety/depression, it has been increasing over the last decade^30^. Another study confirms the preceding fact as they have found only few bipolar affective students compared to students having schizophrenic symptoms^31^. Borderline personality disorder which is the illness of having varying moods has been prevalent in college students, and such students have been in a higher chance of suffering from bipolar disorder^32^. Even though some points out that there is a negative impact for academic performance due to bipolar disorder; work in^33^  suggests that there is not a significant relationship between Grade Point Average (GPA) and bipolar disorder.

Dissociative Disorder (DD) is a psychiatric illness which a person is disconnected from his thoughts, feelings, memories or sense of identity. Dissociative disorder has been proved to be found more in student populations than general populations according to research conducted in^34^. It has been found in non-clinical group of students in Hong-Kong^35^. A study on a group of Italian university students suggests that there is a correlation between dissociation disorder and addiction to internet games^36^. Dissociation and several other factors have led to fear of happiness among college students^37^.

Eating Disorder (ED) is another mental illness that can arise among students. That is having restrictive eating or compulsive eating or irregular or inflexible eating patterns. College student populations have known to be having eating disorders and require treatment as shown in^38^. Eating disorder has been proved to be related to obesity; and weight status has been the predictor of eating disorder symptoms among undergraduates and graduates according to research conducted in^39^. A group of researchers have found that weight motivated vegetarian students have a higher chance of having eating disorders^40^. Nicotine vaping which is common among college students has been found to be correlated with eating disorders^41^. Research conducted using Malaysian university students show that nearly 14% students are suffering from eating disorders^42^. Non-Athletic female students who are dissatisfied with their body shapes have been found to be relatively suffering more from eating disorders^43^. Further, perfectionist female students have shown higher levels of eating disorders as proved in a study conducted among university students^44^. Trait compulsiveness and impulsiveness both have been proved to cause eating disorders in students^45^. Cultural adaptation of dialectical behavioral therapy has been effective in treating an eating disorder of a Chinese student^46^. Some use dissonance-based eating disorder prevention programs to reduce eating disorders among students^47^.

Obsessive Compulsive Disorder (OCD) is a psychiatric illness when a person has uncontrollable, reoccurring thoughts and behaviors that are repeated. A set of college students of Kerala had been found to suffer from this disorder having taboo thoughts and mental rituals as the symptoms^48^. A positive relationship between Orthorexia Nervosa which is an obsession with healthy eating with restrictive behaviors; and obsessive-compulsive disorder has been found among a group of Italian university students^49^. Study shows that fear of COVID-19 has caused university students to get symptoms of OCD^50^. Study suggests that left behind experience as a positive predictor of OCD and suggests improving self-esteem of a person to prevent OCD in students^51^. Hoarding disorder which is the difficulty in getting rid of something had caused OCD in college students^52^. Studies have shown that cognitive behavioral therapy which is a therapy which negative thoughts such as reoccurring thoughts are challenged in order to avoid behavioral patterns such as repeated behaviors; is used to treat OCD^53^.

Schizophrenia is a strong mental illness which people interpret the world abnormally with hallucinations, delusion, disordered thinking and behavior, and lessened emotional expressions. A hallucination is hearing, seeing, smelling, tasting something that does not really exist. Delusions are false beliefs that are not shared by others. It has been found that there is a low probability (0.03) of finding Schizophrenia and Obsessive-compulsive disorder co-occurrence in college students^54^. A study on Japanese university students shows that the number of students who left the schools because of Schizophrenia has been reduced over time^55^. Another study on college students shows that transgender students have a tendency to suffer from schizophrenia more than cisgender female students^56^.

Paranoia is a kind of delusion that the person believes that he/she is threatened by others, even if they aren’t really threatened. Non-clinical group of undergraduates with higher levels of paranoia and anxiety have been found as given in^57^. Research conducted in^58^  shows that high levels of paranoia can be found in students, and investigates on how they experience it. Online imagery has been effective in attenuating paranoia in college students^59^. Mindfulness which is being intensively aware about the thoughts and feelings of the present moment has been effective in attenuating paranoia^60^.

Post-Traumatic Stress Disorder (PTSD) is a mental condition such as memory flashbacks, nightmares or severe anxiety which can occur as a result of an experience of a past traumatic event. A study done on a group of nursing students shows emotional intelligence and psychological resilience as factors contributing to post-traumatic stress growth^61^. Research conducted in^62^  shows that PTSD is found in high school students and concludes that high post-traumatic growth is associated with low frequency use of substances such as alcohol and marijuana. According to the review conducted in^63^ , students with PTSD tend to have a lower IQ, impaired memory, lower verbal abilities, compromised attention thus lowering the academic performance.

Psychosis is a mental state of being detached from reality which the person can experience hallucinations, delusions characterized by agitation and sleep deprivation. Screening tools such as PRIME screen revised and Structured Interview for Psychosis-risk Syndrome (SIPS) have been used in student counseling centers to screen students with psychosis^64^. According to research conducted using a group of British undergraduate students, financial difficulty has been a major contributor to psychosis risk^65^. Fragmented sleep, sleep hallucinations, and night anxiety have been found to correlate with Psychosis like experience in college students^66^. A systematic review done on Psychosis related to students points out that substance use, depression, and younger age as risk factors for psychosis, whereas self-esteem and self-concept as the protective factors^67^. Another research conducted using Chinese students shows that social support and resilience can act as protective factors for psychosis in students^68^. Students with Psychosis tend to show poorer cognitive functions than normal students according to research conducted in^69^.

### Motivation

A recent study on the overall mental well-being of medical undergraduates of Sri Lanka points out that around 40% are in severe psychological distress^70^. Another study in Sri Lanka done among nursing students shows the evidence of presence of depression, anxiety, and stress among them^71^. There is evidence to prove burnout in high school students due to being disturbed while studying, and due to being bullied in school^72^. Recent studies suggest that students’ stress level has been increased during the online learning and evaluation due to the COVID-19 pandemic^73^. Since the pandemic still exists at the time of conducting this research; it is more probable to find psychiatric illnesses among students in higher education. Preceding works fail to address how different mental illnesses exist in both clinical and non-clinical undergraduates comprehensively.

It is yet unknown which academic components cause mental illnesses such as GAD, depression in students. Most of the existing literature addresses anxiety, depression, eating disorders of students; but other potential mental illnesses such as schizophrenia, agoraphobia, paranoia, post-traumatic stress are least addressed in literature. In case of post-traumatic stress, it is unknown whether ragging has caused post-traumatic stress disorder in students, even though a case study on university of Colombo has found ragging as a cause of stress among undergraduates^74^. There is no evidence to prove whether mental illnesses such as schizophrenia exist among university students in Sri Lanka or not. Most researchers have studied on medical students, and it is not known how mental illnesses are spread in other disciplines.

### Problem statement

The research problem is the lack of comprehensive study on the mental health of Sri Lankan students in higher education which shows the distribution of such diagnosed students under different mental illnesses under diverse factors, and lack of knowledge on the universities’/students’ interventions to reduce the negative effects of such mental illnesses.

### Objectives

- To identify the prevalence level of 13 mental illnesses among university students of Sri Lanka;
- To identify the overall psychological and social well-being of students in Higher education in Sri Lanka;
- To investigate whether the universities/students have remedies to prevent or reduce the mental illnesses of the students and its effectiveness;
- To identify the demographic factors, academic stressors, environmental stressors contributing for the prevalence of each psychiatric illness.

### Benefits to community and social value

The research findings will play a vital aspect in determining the requirement to implement psychological distress reduction/prevention programs in universities. Since this research identifies the factors and components creating stress in students, academic programs can be designed by targeting the aspects which cause stress in order to reduce or prevent them. In addition, this work identifies the correlation between a set of demographic factors, academic factors, and environmental factors for the prevalence of each psychiatric illness and psychiatric illnesses in general. The identified risk factors will help policy makers of the university to design curriculum to reduce the risk factors, expand resources to reduce the risk. For example, according to our results, as the majority of students have felt the highest stress for the online written examination (AS3) and it has been identified as a risk factor, the policymakers should avoid conducting online written examinations as much as possible. In this manner, identification of risk factors will help policymakers of university to plan the delivery mode of the curriculum in order to reduce the risk of development of psychiatric illnesses among students. It is very beneficial for the society to identify the prevalence level of mental disorders, as non-diagnosed mental disorders can negatively affect educational institutions, the safety of the individual, and society that the psychiatrically ill individual interact with. Based on the results of this study, the policymakers may direct the students to seek the advice of psychiatrists to reduce the negative effects of the illnesses. By timely visiting of a psychiatrist for proper medical advice and medications for those students who are severely ill, both individual and the society can be benefitted. The academic performance of students who seek proper medical advice for their psychiatric illness can be expected to be improved, as many researches reviewed in the literature review have found out that psychiatric illnesses degrade the academic performance of students. By participating in the survey, the students will engage in capacity building of their knowledge regarding their own psychological well-being. They can keep a copy of the responses to self-evaluate them. Hence, both the students and institutions will be benefited from this research. By producing graduates with high emotional well-being, the society will be benefited.

### Benefits to healthcare professionals

As this research investigates on factors causing psychological distress among students, knowing these factors will help healthcare professionals in treating students diagnosed with a particular psychiatric illness. Identification of the risk factors will help psychiatrists in providing special treatments for the psychiatric illnesses. For example, our study proves that anxiety related to written end examination causes SAD. When the psychiatrist knows about this, he may prescribe a behavioral therapy to reduce the anxiety level related to the examination. Therefore, when the psychiatrist is already aware about the factors causing the psychiatric illness, it will be helpful for the psychiatrist in treating the patient. The research may reveal hidden/not revealed factors/knowledge so far about the prevalence of psychiatric illnesses among students such as accommodation problems which other researchers have not yet investigated.

## Material and methods

### Ethical approval and adherence to guidelines and regulations

Ethical approval was obtained from the ethics review committee of University of Ruhuna and from the vice chancellor of university of Ruhuna in writing before the data collection. All experiments (data collection using the questionnaire) were performed in accordance with relevant guidelines/regulations (declaration of Helsinki, SAGER guidelines). Informed consent was obtained from all the participants before the data collection. All participants’ responses were collected in anonymous mode and participants’ identifying information were not collected. There are no legal/social/financial issues for this research.

### Sample

#### Scope and time period

This research is done for the whole university since data collected from a single faculty will not provide a broad understanding about the psychological state of the Sri Lankan students in higher education. Specifically, we attempt to eliminate the data set bias by a whole university study. Further, some psychiatric illnesses may be rare. By increasing the number of students for which the questionnaire is distributed, we increase the probability of finding such rare cases. The study was conducted among the undergraduates of university of Ruhuna in the period from 15th of December 2021 to 10th of January 2022.

#### Confidentiality and anonymity

A response by a student does not include any personal information such as student name, student identification numbers, etc. We do not even collect the name of the faculty which the student is studying. Hence, all responses will be anonymous. The collected data will be confidentially kept (stored in Google Drive without sharing) with the principal investigator forever. Participants cannot be provided with any incentive/reward due to anonymity.

#### Participants

The students participate for the survey in their own willingness. They have the right to not respond to the questionnaire. However, if they submit a response; that response cannot be withdrawn. That is because by providing a complete response, they have agreed and provided consent to participation for the research. At the beginning of the survey, a participant must provide consent to participate in the survey, and agree that he/she has read and understood about information collected in the survey by ticking a checkbox. The information presented to the participant are purpose of the survey, participant’s responsibilities, potential benefits and risks to the participant, confidentiality and anonymity of data collection, and process and period of storage of data. A participant has the right to contact the principal investigator via email in case of any difficulty regarding the survey. The participants cannot be informed about the results, as contact details of the participants are not collected.

#### Inclusion and exclusion criteria

All responses from Sri Lankan undergraduates who have finished their degree within the last 2 years or currently enrolled in a degree program are included. That is because, including responses from old graduates may not provide correct insight about current situation of the psychological condition of the students in higher education. Further, we only accept full responses as we have set settings in Google forms to exclude partial responses automatically. We do not exclude the students who are already diagnosed with a psychiatric illness by a psychiatrist, since this survey is to know about students with psychiatric illnesses. Either the student is diagnosed by self-reporting or diagnosed by a psychiatrist; it does not matter.

#### Contact method

The participants are contacted through head of the departments in faculties. An electronic mail is written to the head of the department, requesting to distribute the questionnaire among all students belonging to the department.

#### Sample size and response rate

The sample consists of total responses screened with inclusion and exclusion criteria. 100 responses were received, and the total number of responses after screening were also 100, since all responses satisfied the inclusion criteria. Therefore, the response percentage was 1.00%, since university of Ruhuna has 10, 000 undergraduates.

### Data collection tool

The data collection tool is a comprehensive questionnaire consisting of 19 sections implemented and shared across universities in the form of a Google form. Each of the section of the questionnaire is as listed below.

- Section 1 collects the demographic and academic information about the student such as gender, age, civil status, ethnicity, religion, academic year, Overall Grade Point Average (OGPA), expected class of the degree, total family income, and residence.
- Section 2 collects information about already diagnosed mental illnesses and remedies from students and the university.
  - First a question is asked as “Have you been ever diagnosed with a psychiatric illness by a psychiatrist?”. If the student responds yes; he/she will have to answer a set of more questions.
  - Next, the time of diagnosis, whether it was before the commencement of the degree program or during the degree program will be asked.
  - After that, he/she will be asked to tick the names of all mental illnesses that he/she had been already diagnosed with. Then, a list of remedies is presented to the student to tick the remedies that is done by the student for the diagnosed illness. Remedies provided are medicine, mindfulness meditation, cognitive behavioral therapy, biofeedback, animal assisted therapy, relaxation techniques (music, sports, leisure activities), support from family and friends, cultural adaptation of dialectical behavioral therapy, drinking alcohol, smoking nicotine or marijuana, watching porn, dissonance-based eating disorder prevention, online social networks, and online gaming.
  - Next, the question “Select all the support received from the university to treat your psychiatric illness” will be asked. Here, a list is given and in addition the student can specify any other support given. The contents of the list are counseling services, cultural events organizing, providing resources for sports, music, etc., financial support, multi-stage testing, None, and other.
  - Finally, we ask the student to self-evaluate the effectiveness of the students/universities remedies in treating the psychiatric illness in a scale of 1–5.
- Section 3 collects information about overall distress scale of the student to assess the likelihood of having a mental disorder. For this purpose, we use the Kessler Psychological Distress Scale (KPDS) which is a collection of 10 questions to measure the level of distress of a person^75^. A response has a total mark (T) ranging from 10-50, and the distress level is decided as given in Eq. 1.
  1 $$\begin{aligned} Distress \,level = \left\{ \begin{array}{ll} Normal &{} if\, 10\, \le \, T\, \le \,19 \\ Mild\,Distress\, &{} if\, 20\, \le \, T\, \le \,24\\ Moderate\,Distress\, &{} if\, 25\, \le \, T\, \le \,29\\ Severe\,Distress &{} if\, 30\, \le \, T\, \le \,50\\ \end{array} \right. \end{aligned}$$
- Section 4 collects information about the emotional well-being (question 1–3), social well-being (question 4–8), and psychological well-being (question 9–14) of the student using the Mental Health Continuum - Short Form (MHC-SF)^76^  which consists of 14 questions. The total score (T) for a response ranges from 0 to 70, and the well-being class is classified given in Eq. 2.
  2 $$\begin{aligned} Well-being \,level = \left\{ \begin{array}{ll} Moderate &{} if\, 0\, \le \, T\, \le \,14 \\ Languishing\, &{} if\, 15\, \le \, T\, \le \,42\\ Flourishing\, &{} if\, 43\, \le \, T\, \le \,70\\ \end{array} \right. \end{aligned}$$
- Section 5 collects information about the academic components which stress the students. The first question is “In a scale of 1–5 how much are you satisfied about the degree program?” - AS1 to know the overall satisfaction about the degree program. Next, we present academic components for the students to rate the level of stress in a scale of 1–5. The Academic Stressors (AS) provided are;
  - Conventional written end semester/year end examination - AS2
  - Online written end semester/year-end examination -AS3
  - Oral examination (viva) - AS4
  - Oral presentation - AS5
  - Individual self-learning (your own studies) - AS6
  - Participation for a physical lecture - AS7
  - Participation for an online lecture - AS8
  - Active learning (debates/case studies/small group discussions/role-plays etc.) involving group work - AS9
  - Research and project development work - AS10
  - Online quiz - AS11
  - Physical in-class tests - AS12
  - Practical demonstrations - AS13
  - Industrial/Professional/Worksite training - AS14
  If any academic component is not relevant to them, they can select the option “Not Applicable”.
- Section 6 is on collecting information about environmental factors contributing to academic stress known as Environmental Stressors (ES). We provide a set of environmental factors, and ask the student to mark the level of stress associated with each factor, in a scale of 1-5. If the environmental factor is not relevant to them, they can select the option “Not Applicable”. The environmental factors given are;
  - Prevalence of financial difficulties - ES1
  - COVID19 - ES2
  - Presence of a physical illness - ES3
  - Relationship problems - ES4
  - Bad experience due to ragging - ES5
  - Death/Sickness of a close associate - ES6
  - Accommodation problems - ES7
  - Problems in the teaching-learning process - ES8
  - Troubles in online learning and evaluation - ES9
  - Lack of support for psychiatric help - ES10
  - Having less/no time to spend for leisure/sports/music etc. - ES11
  Last question is asking the student to specify if there are any other environmental stressors.
- Section 7 collects information about GAD. First, the reader is explained with the definition of GAD. We use the Anxiety Symptom Questionnaire (ASQ)^77^  with each response per question having a 2-fold scale (’Yes’ or ’No’). ASQ questions 9–11 are used in order to screen for GAD, as given in Eq. 3.
  3 $$\begin{aligned} GAD \,class = \left\{ \begin{array}{ll} GAD\,possibile\, &{} if\,only\,question \,9\,and\,10\, marked\\ Mild\,GAD\, &{} if\,all\,questions \, marked\,with\,1-2\,symptoms\,for\,question\,11\\ High\,GAD &{} if\,all\,questions\, marked\, with\, more\,than\,2\, symptoms\, for \,question \,11\\ Normal &{} if\, otherwise \\ \end{array} \right. \end{aligned}$$
- Section 8 collects information about Agoraphobia. First, the reader is explained with the definition of Agoraphobia. We use ASQ questions 5–6 in order to screen for Agoraphobia, as given in Eq. 4.
  4 $$\begin{aligned} Agoraphobia \,class = \left\{ \begin{array}{ll} Possible\, &{} if\,only\,question \,5\, marked\\ Mild\,agoraphobia &{} if\,all\,questions\, marked\, with\, 1-2\, symptoms\, for \,question \,6\\ Moderate\,agoraphobia &{} if\,all\,questions\, marked\, with\, 3-4\, symptoms\, for \,question \,6\\ Severe\,agoraphobia &{} if\,all\,questions\,marked\,with\, more\,than\,4\,symptoms\,for\,Q6.\\ Normal &{} if\,otherwise \\ \end{array} \right. \end{aligned}$$
- Section 9 collects information about Panic Disorder (PD). First, the reader is explained with the definition of PD. We use ASQ questions 1–4 in order to screen for PD, as given in Eq. 5.
  5 $$\begin{aligned} Panic\, Disorder \,class = \left\{ \begin{array}{ll} Not\,necessarily\, &{} if\,only\,question \,1\, and\,2\, marked\\ Panic\, attack\, possible &{} if\,only\,question \,1,\,2\, and\,3 \, marked\\ Panic\,attack\,exist &{} if\,4\,or\,more\, symptoms\,for\,Q4 \\ Normal &{} if\,otherwise \\ \end{array} \right. \end{aligned}$$
- Section 10 collects information about SAD. First, the reader is explained with the definition of SAD. We use ASQ questions 7–8 in order to screen for SAD, as given in Eq. 6.
  6 $$\begin{aligned} SAD \,class = \left\{ \begin{array}{ll} Possible\, &{} if\,only\,question \,7\, marked\\ Mild\,SAD &{} if\,all\,questions\, marked\, with\, 1-2\, symptoms\, for \,question \,8\\ Moderate\,SAD &{} if\,all\,questions\, marked\, with\, 3-4\, symptoms\, for \,question \,8\\ Severe\,SAD &{} if\,all\,questions\,marked\,with\, more\,than\,4\,symptoms\,for\,Q8.\\ Normal &{} if\, otherwise \\ \end{array} \right. \end{aligned}$$
- Section 11 collects information about depression. First, the reader is explained with the definition of depression. Beck’s Depression Inventory (BDI-21)^78^ , which is a questionnaire consisting of 21 questions with each response per question having a 4-fold scale, is used to screen depression. A response can have a total score (T) ranging from 0 to 63, and the scores are classified into depression classes, as given in Eq. 7.
  7 $$\begin{aligned} Depression\,class = \left\{ \begin{array}{ll} Normal &{} if\, T\, \le \,10 \\ Mild\,mood\, disturbance &{} if\,11\, \le \, T\,\le 16 \\ Borderline\, clinical\, depression &{} if\,17\, \le \, T\,\le 20 \\ Moderate\, depression &{} if\,21\, \le \, T\,\le 30 \\ Severe\, depression &{} if\,31\, \le \, T\,\le 40 \\ Extreme\, depression&{} if\,41\, \le \, T\, \\ \end{array} \right. \end{aligned}$$
- Section 12 collects information about BAD. First, the reader is explained with the definition of BAD. Mood Disorder Questionnaire (MDQ)^79^  consisting of 17 questions with each response per question having a 2-fold (’Yes’ or ’No’) answer, is used to screen the students suffering from BAD, as given in Eq. 8.
  8 $$\begin{aligned} BAD \,class = \left\{ \begin{array}{ll} BAD\,present &{} if\,more\,than\,6\,yes\,for\, Q1-13,\,yes\,for\,Q14\, \& \,moderate/serious\,for\,Q15\\ Normal &{} if\,otherwise \end{array} \right. \end{aligned}$$
- Section 13 collects information about DD. First, the reader is explained with the definition of DD. Somatoform Dissociation Questionnaire (SDQ) consists of 20 questions with each response per question having a 5-fold scale. We obtain the 5-question version, which is the questions 4, 8, 13, 15, and 18 of the original SDQ-20, as SDQ-5^80^  to screen the students suffering from DD. The total score (T) for a response for SDQ-5 ranges from 0-25. The screening criteria for DD is as given in Eq. 9.
  9 $$\begin{aligned} DD\,class = \left\{ \begin{array}{ll} DD\,present &{} if\,8\, \le \, T\, \\ \\ Normal &{} if\,otherwise \end{array} \right. \end{aligned}$$
- Section 14 collects information about ED. First, the reader is explained with the definition of ED. Eating Disorder Examination - Questionnaire Short (EDE-QS)^81^  consisting of 12 questions with each response per question having a 4-fold scale, is used to screen the students suffering from ED. The total score (T) ranges from 0-36, and the screening criteria for ED is given in Eq. 10.
  10 $$\begin{aligned} ED\,class = \left\{ \begin{array}{ll} Normal &{} if\, T\, \le \,7 \\ Mild\,ED\, &{} if\,8\, \le \, T\,\le 14 \\ Moderate\, ED &{} if\,15\, \le \, T\,\le 21 \\ Severe\, ED &{} if\,22\, \le \, T\,\le 28 \\ Extreme\, ED&{} if\,29\, \le \, T\, \\ \end{array} \right. \end{aligned}$$
- Section 15 collects information about OCD. First, the reader is explained with the definition of OCD. We use ASQ questions 12–13 in order to screen students with OCD, using the criterion given in Eq. 11.
  11 $$\begin{aligned} OCD \,class = \left\{ \begin{array}{ll} OCD\,present\, &{} if\,either\,or\,both\,questions\, marked\\ Normal &{} if\,otherwise\\ \end{array} \right. \end{aligned}$$
- Section 16 collects information about schizophrenia. First, the reader is explained with the definition schizophrenia. Functional Remission of General Schizophrenia (FROGS) Scale has 19 questions with each response per question having a 5-fold scale. We use the 4-item mini-FROGS scale^82^  in order to screen students with schizophrenia. A response contains a total mark (T) ranging from 0-16, and the presence of Schizophrenia is decided as given in Eq. 12.
  12 $$\begin{aligned} Schizophrenia \,class = \left\{ \begin{array}{ll} Schizophrenia\,present\, &{} if\, 8 \le T\\ Normal &{} if\,otherwise\\ \end{array} \right. \end{aligned}$$
- Section 17 collects information about paranoia. First, the reader is explained with the definition paranoia. Paranoia Worries Questionnaire (PWQ)^83^  having 5 questions with each response per question having a 5-fold scale, is used in order to screen students with paranoia. The total score (T) of a response range from 0-20, and the presence of paranoia is decided, as given in Eq. 13.
  13 $$\begin{aligned} Paranoia \,class = \left\{ \begin{array}{ll} Paranoia\,present\, &{} if\, 5 \le T\\ Normal &{} if\,otherwise\\ \end{array} \right. \end{aligned}$$
- Section 18 collects information about PTSD. First, the reader is explained with the definition PTSD. We use ASQ questions 14–17 in order to screen students with PTSD as shown in Eq. 14. We ask an additional question ’Was ragging a traumatic event for you?’.
  14 $$\begin{aligned} PTSD \,class = \left\{ \begin{array}{ll} PTSD\,present &{} if\,Q14\,marked\,with\, minimum\,1,3,2\,symptoms\,for\,Q15,Q16,Q17\,respecti.\\ Normal &{} if\,Otherwise\\ \end{array} \right. \end{aligned}$$
- Section 19 collects information about psychosis. First, the reader is explained with the definition psychosis. Prodromal Questionnaire (PQ)^84^  having 16 questions with each response per question having a 4-fold scale, is used in order to screen students with psychosis. A response conveys a total mark (T) ranging from 0 to 48, and the psychosis classes are derived, as given in Eq. 15.

15 $$\begin{aligned} Psychosis\,class = \left\{ \begin{array}{ll} Normal &{} if\, T\, \le \,11 \\ Mild\,psychosis\, &{} if\,12\, \le \, T\,\le 23 \\ Moderate\, psychosis &{} if\,24\, \le \, T\,\le 35 \\ Severe\, psychosis &{} if\,36\, \le \, T \\ \end{array} \right. \end{aligned}$$

### Data analysis tool

For the data analysis, we use Microsoft Office Excel 2016 to store and analyze data. A third-party Excel Add-on called Real Statistics is used to perform binary logistic regression for predicting the prevalence of each of the psychiatric illnesses using demographic information, academic stressors, and environmental stressors. If a given stressor or demographic factor contribute positively for the presence of more than 7 psychiatric illnesses, we categorize such factors as risk factors. The risk percentage is calculated as given in Eq. 16.

16 $$\begin{aligned} \begin{aligned} Risk\, perce.\, (R) = \frac{100*(No. of BLR\, coefficient\, positive\,illnesses)}{Total\,psychiatric\, illnesses} \\ \end{aligned} \end{aligned}$$

### Ethical approval and consent to participate

The present study was approved by the Ethics Review Committee of University of Ruhuna and by the Vice Chancellor. All participants gave their informed consent for participation in the study before filling in the questionnaire.

## Results

### Population characteristics of the sample

The mean age of the participants was 23.64 with a standard deviation of 1.40. The mean OGPA of the participants was 2.93 with a standard deviation of 0.69. Other population characteristics are summarized in Table 1. When the collected data is analyzed statistically, the population characteristics are encoded as given in brackets of each subdivision of the characteristic. Age is received in its raw form.

**Table 1:** Population characteristics distribution of the collected sample of students.

Population characteristic	Subdivision (encoding)	Number (percentage)
Gender	Male (1)	67 (67)
	Female (0)	33 (33)
Civil status	Unmarried (0)	99 (99)
	Married (1)	1 (1)
Ethnicity	Sinhala (0)	88 (88)
	Tamil (1)	7 (7)
	Moor (2)	4 (4)
	Other (3)	1 (1)
Religion	Buddhist (0)	83 (83)
	Christian (1)	8 (8)
	Muslim (2)	3 (3)
	Hindu (3)	6 (6)
Academic year	Year 1 (0)	16 (16)
	Year 2 (1)	8 (8)
	Year 3 (2)	57 (57)
	Year 4 (3)	17 (17)
	Year 5 (4)	0 (0)
	completed (5)	2 (2)
Degree class	Normal hons. (0)	19 (19)
	Second lower (1)	16 (16)
	Second upper (2)	40 (40)
	First class (3)	25 (25)
Total family income	0–10000 (0)	3 (3)
	20000–30000 (1)	19 (19)
	30000–40000 (2)	11 (11)
	40000–50000 (3)	10 (10)
	50000–60000 (4)	12 (12)
	60000–70000 (5)	12 (12)
	70000–80000 (6)	6 (6)
	80000–90000 (7)	3 (3)
	90000–100000 (8)	5 (5)
	greater than 100000 (9)	19 (19)
Residence	Inside the university (0)	78 (78)
	Outside rented place (1)	7 (7)
	From home (2)	15 (15)

### Already diagnosed students

Only 8% of the students are already diagnosed with a psychiatric illness by a psychiatrist. 87% of the students those who were diagnosed with a psychiatric illness have been diagnosed during the degree program. They have been diagnosed with GAD, PD, SAD, depression, ED, OCD, PTSD, and psychosis. They have not been diagnosed with other psychiatric illnesses (5 remaining psychiatric illnesses) considered in this research.

### Remedies undertaken by students to reduce psychological distress

**Figure 1:**
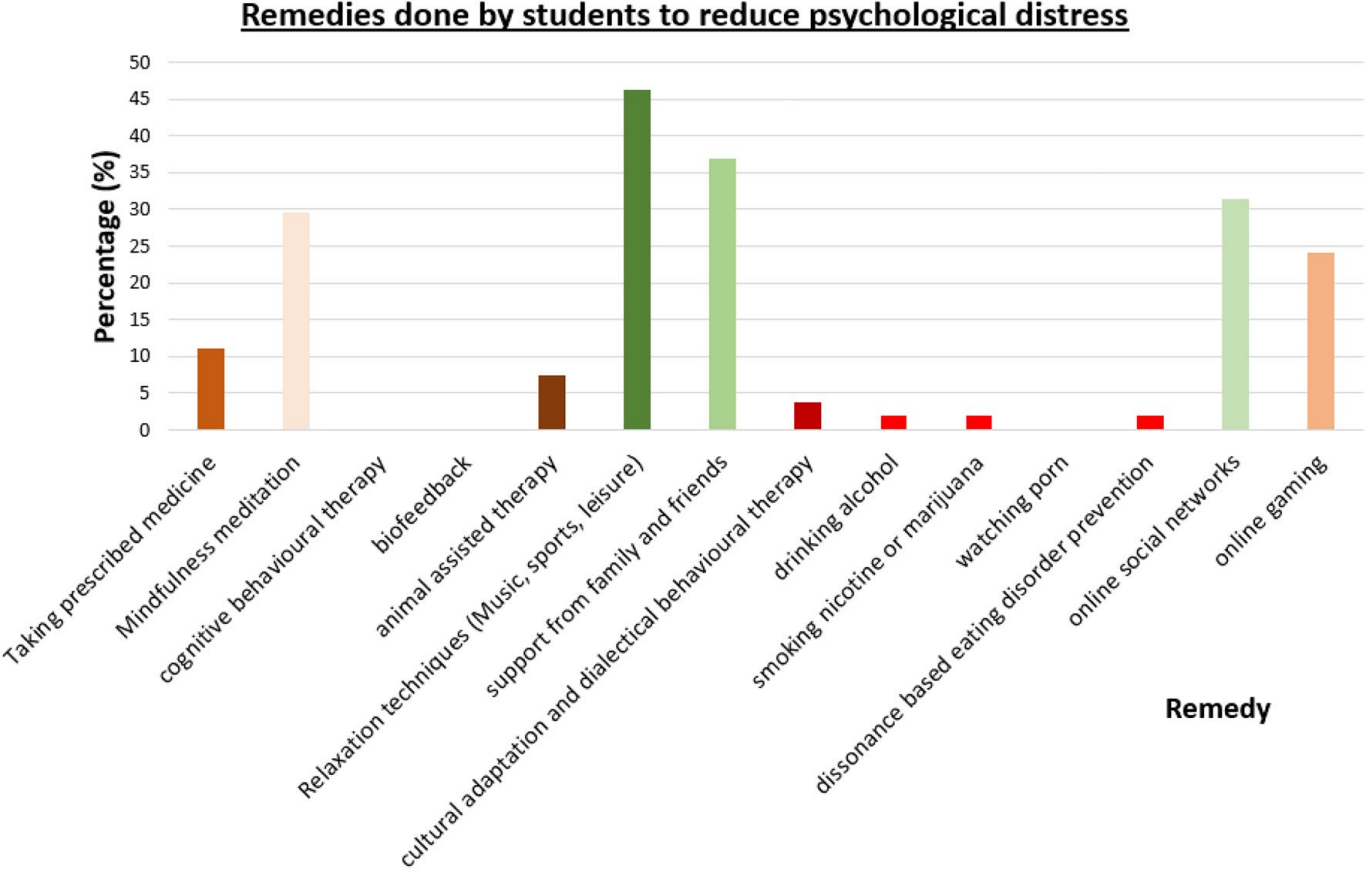
Distribution of percentage of students applying the remedies to reduce psychological distress.

The responses for the remedies taken by students to reduce the psychological distress were collected, and the percentage of students applying the remedy is plotted as shown in Fig. 1.

As evident from the Fig. 1, most frequently used remedy is relaxation techniques (Music, sports, leisure, etc.) followed by support from family and friends, online social networks, mindfulness meditation, and online gaming. A significant percentage of students take the prescribed medicine for the psychiatric illness, and use animal assisted therapies. Other remedies in Fig. 1 are either not used by students or insignificant.

### Supports received from the university to treat psychiatric illnesses

According to the responses, 61% of the students mention that they don’t receive psychiatric help from the university. The dominant support received from the university has been providing resources for sports, music followed by cultural event organizing, counseling services, and financial support according to the responses received from the students. Further, 26% of the students mention that the supports received from the university are highly effective in treating their psychiatric illnesses.

### Psychological distress

**Figure 2:**
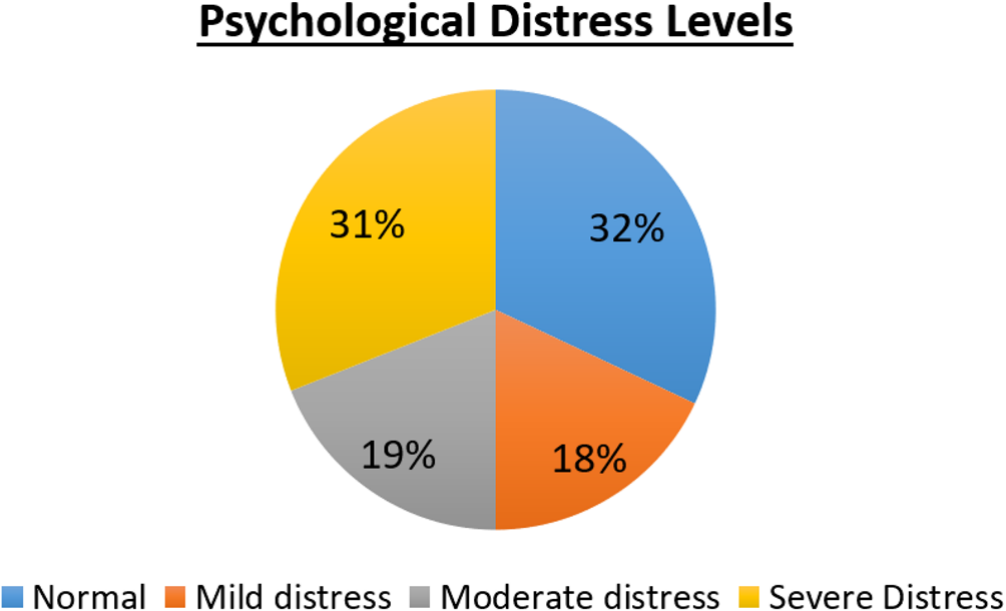
Psychological distress distribution among university students.

The responses for the KPDS were numerically encoded, and analyzed. The total score for KPDS had a mean value of 25.07, and a standard deviation of 8.79. The students’ psychological distress were classified as shown in Fig. 2.

As seen from Fig. 2, only 32% of the students are Normal (not psychologically distressed). Around two thirds of the student population is psychologically distressed and 50% of the students are moderately to severely distressed. This result can be interpreted as a warning sign for the students, as they show a high potential for psychiatric illnesses due to the distress.

### Emotional, social, and psychological well-being

Even though the students were proved to be psychologically distressed in the previous section, they have scored satisfactorily high scores for the MHC-SF, which assesses the emotional, social, and psychological well-being of the students. The total score for MHC-SF had a mean value of 33.09, and a standard deviation of 14.18. According to the responses, 10% of the students have moderate well-being, 66% have languishing well-being, and 24% have flourishing well-being. This can be argued as because of the remedies/precautionary measures undertaken by the students to reduce the psychological distress. So, even though they are stressed, they have been able to maintain emotional, social, and psychological well-being satisfactorily.

### Academic components which stresses the students

**Figure 3:**
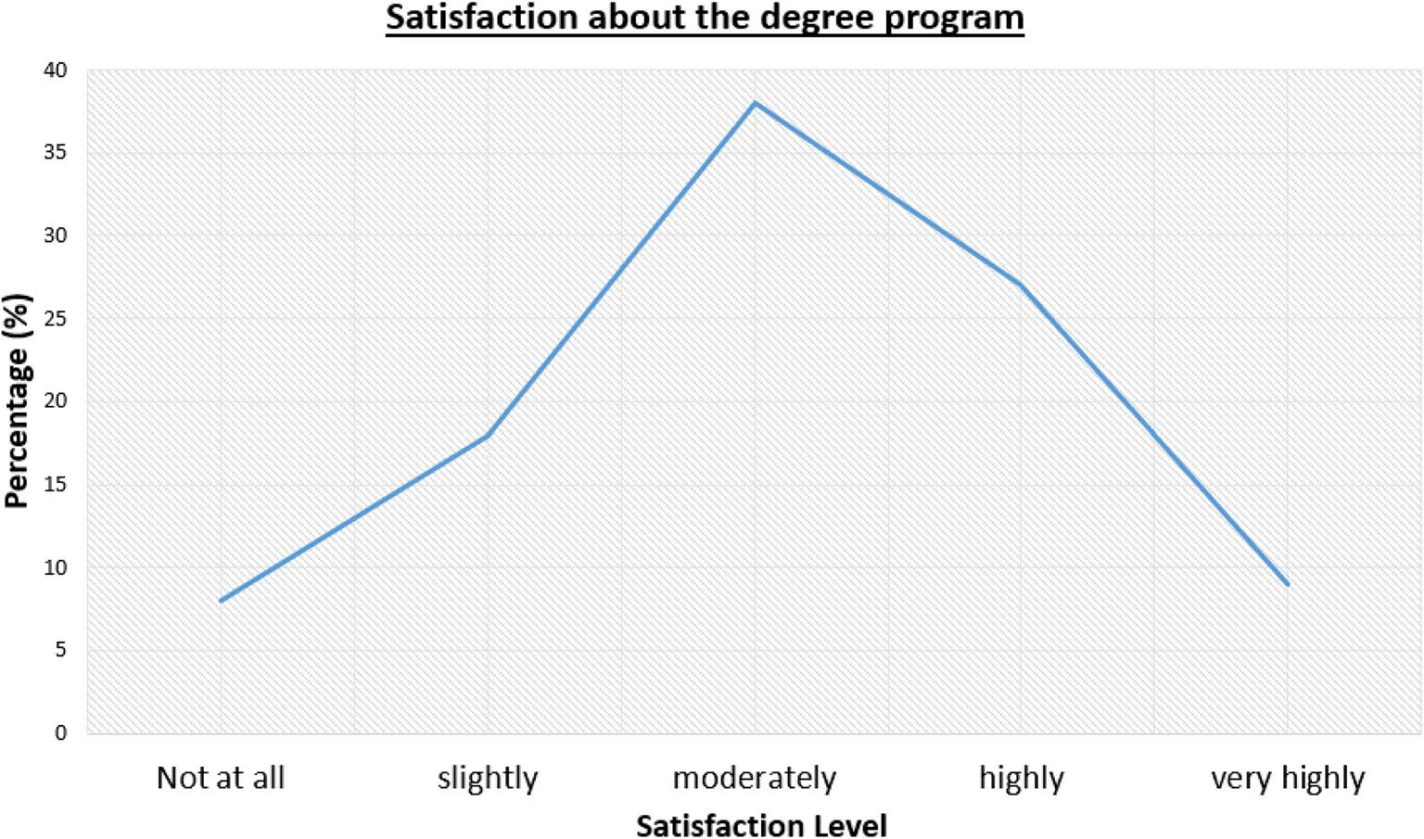
Satisfaction about the degree program distribution among university students.

The overall satisfaction about the academic program is as follows. 8% of the students were not satisfied at all on the degree program, while 18% were slightly satisfied. 38% were moderately satisfied, 27% were highly satisfied, and 9% were very highly satisfied. Therefore, a slightly positively skewed bell-shaped distribution of satisfaction with the degree program can be observed, as evident from Fig. 3.

**Figure 4:**
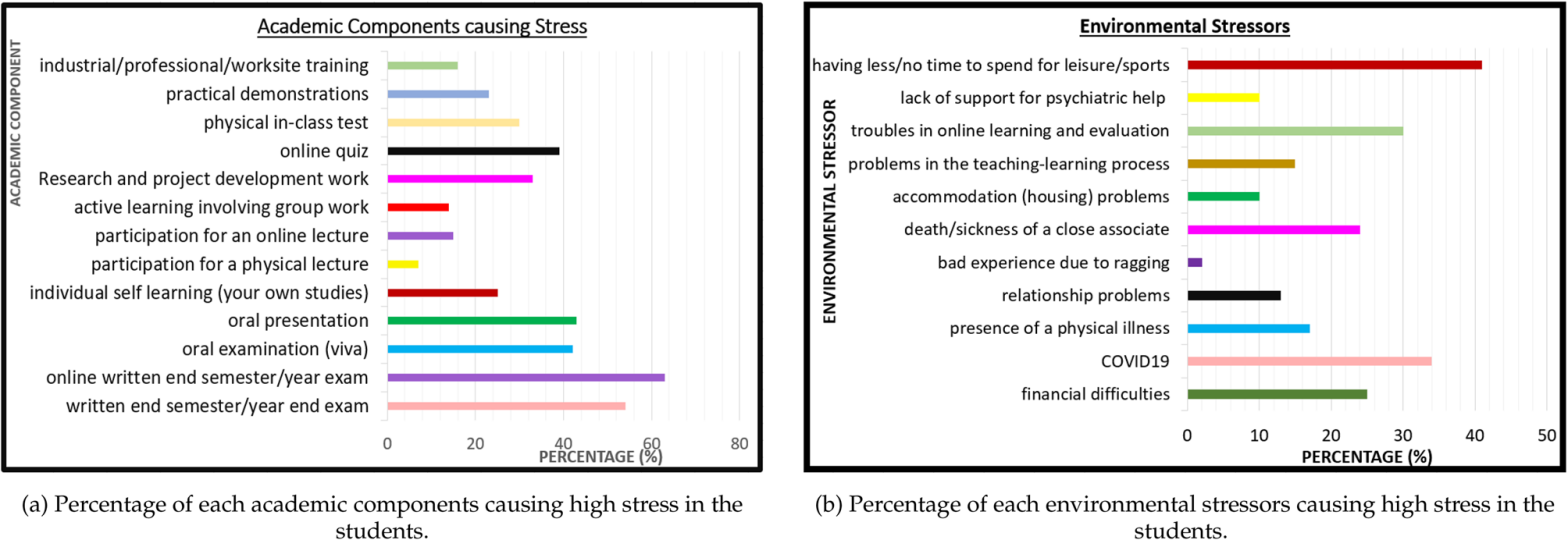
Percentage of academic and environmental stressors causing stress in students.

In order to understand about the academic components which stress the students, we extract the percentage of students who responded as either highly or very highly stressed for each academic component, and plot as shown in Fig. 4a.

As evident from the graph in Fig. 4a, the highest stress is caused by written end semester or year-end exams, where the online mode being more stressful than the conventional mode. The next level of high stress can be found in oral examinations followed by online quizzes, in-class tests, and research and development work. On the other hand, learning tasks such as participation for lectures and active learning, cause minimum levels of stress in the students. Worksite training and practical demonstrations seem to cause an intermediate level of stress compared to other academic components.

### Environmental factors contributing to academic stress

In order to understand about the environmental stressors which stress the students, we extract the percentage of students who responded as either highly or very highly stressed for each environmental stressor, and plot as shown in Fig. 4b.

Due to the academic workload, having less/no time to spend for leisure/sports/music etc. has been the dominant environmental stressor, as evident from Fig. 4b. Next highest stressor has been COVID-19 pandemic, followed by troubles in online learning and evaluation, which is again a consequence of COVID-19. This proves that COVID-19 and online learning have increased the stress levels in students. Surprisingly, financial problems, death of a close associate, presence of a physical illness, etc. cause intermediate levels of stresses in students. Students report that they feel low levels of stress for relationship problems, accommodation problems, and bad experience due to ragging. So, in conclusion, students have felt highly stressed for stressors which are directly associated with teaching-learning process, and vice versa. Further, students report that they feel stressed in following situations.

- Noisy surrounding while studying.
- Language problems.
- Insufficient food quality and the higher price.

### Screening for psychiatric illnesses

In this section, we will screen the students for prevalence of each of the psychiatric illness, as described by the following subsections.

#### Screening for GAD

**Figure 5:**
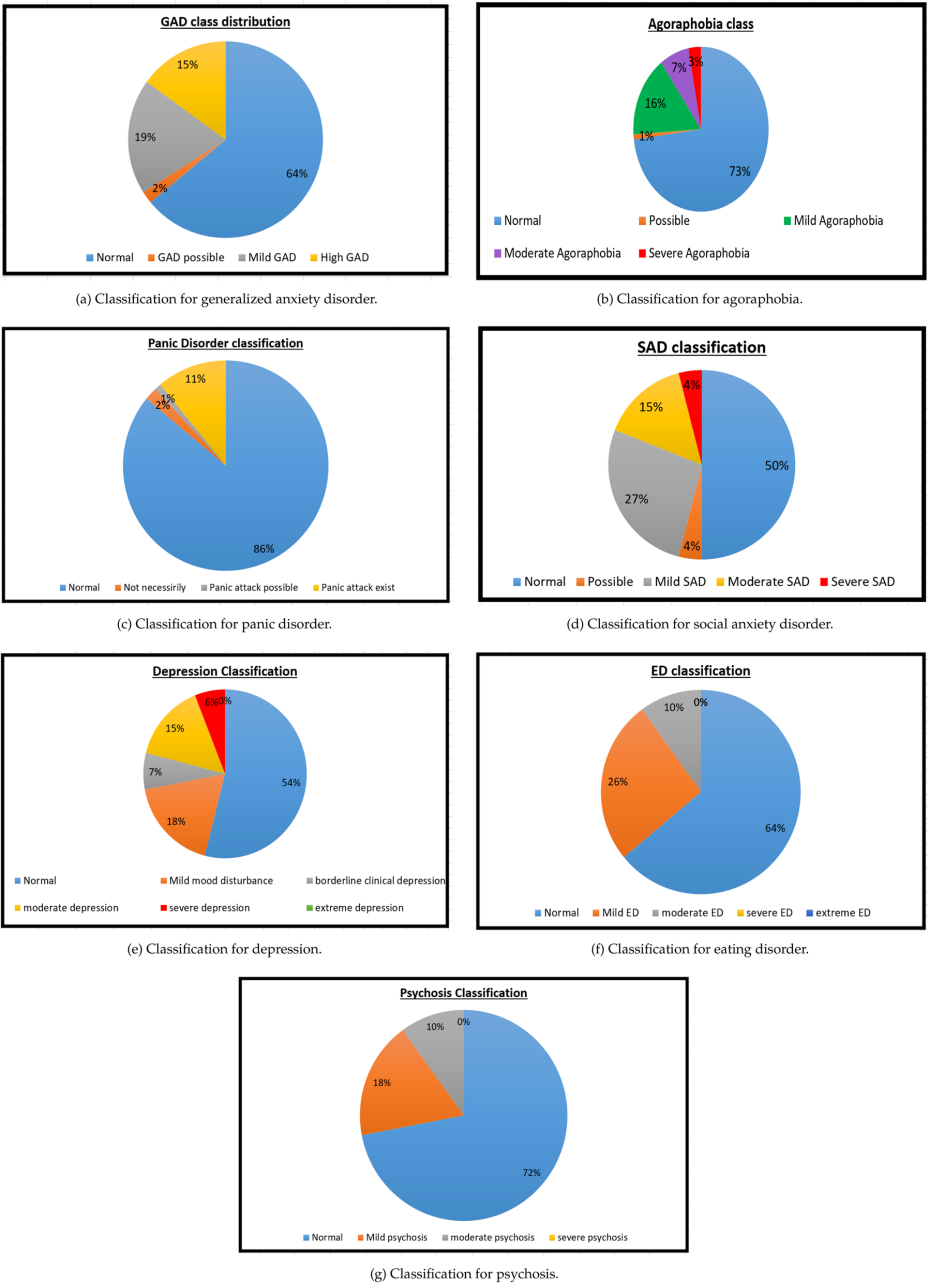
Screening results for different types of psychiatric illnesses.

The responses for ASQ questions 9–11 were encoded with numerical values, and analyzed. The results are as shown in Fig. 5a.

According to the results, nearly two thirds of the student population have been classified as Normal with respect to GAD, as evident from Fig. 5a. Out of the remaining one third, most students show symptoms for Mild GAD, and 42% show high GAD symptoms. Only a few (2%) are categorized with the possibility to have GAD. Therefore, a significant fraction (0.15) of the population of the students has high GAD.

#### Screening for agoraphobia

The responses for ASQ questions 5–6 were encoded with numerical values, and analyzed. The results are as shown in Fig. 5b.

As evident from the Fig. 5b, around one fourths of the student population show agoraphobia symptoms. Out of the students who show agoraphobia symptoms, most show mild symptoms, while 37% (10% of the whole population) show moderate to severe symptoms. Only 1% of the population showed the possibility to have agoraphobia.

#### Screening for PD

The responses for ASQ 1-4 were encoded with numerical values, and analyzed in order to obtain the classes of PD as shown in Fig. 5c.

Panic disorder can be found in 11% of the student population, as evident in Fig. 5c. There are also few (3%) students who can be possibly having a panic attack. But the majority of the student population do not have PD.

#### Screening for SAD

Students were screened for SAD using ASQ 7-8. The responses received were encoded with numerical values, and classified into SAD classes after response analysis, as shown in Fig. 5d.

Half of the entire student population suffers from SAD according to the screened results in Fig. 5d. Out of the population suffering from SAD, only 8% show severe SAD symptoms, and majority are either having mild or moderate symptoms.

#### Screening for depression

The responses of students for BDI-21 were encoded with numerical values, and analyzed. The results are given in Fig. 5e. The student response “prefer not to answer” for the question regarding interest towards sex was encoded as value 1.5. Justification for doing so is to provide an intermediate response, without considering it as a response between the two ends of having no change in interest towards sex (encoded 0), and having lost interest towards sex completely (encoded as 3).

The mean score for the BDI-21 was 12.05, with a 95% confidence interval of [0, 31.91]. 54% of the student population were screened as normal, while 7% were found with borderline clinical depression, and 18% with mild mood disturbance. Therefore, only 21 % of the responses show either moderate or severe depression, while there were no cases for extreme depression. This proves that nearly one fifth of the student population have clinical depression symptoms.

#### Screening for BAD

The responses for MDQ were encoded into numerical values, and analyzed to obtain the classification for BAD. The analyzed responses show that 8% of the student population have BAD, while 92% do not have the disorder.

#### Screening for DD

The responses for SDQ were encoded into numerical values, and analyzed. The mean score for the responses was 2.05, with a 95% confidence interval of [0, 8.37]. According to the analysis, 11% of the student population showed symptoms for DD, while 89% were normal with respect to the DD.

#### Screening for ED

The responses for EDE-QS were encoded, and analyzed to obtain the classification for ED as given in Fig. 5f. The mean score for the EDE-QS was 6.51, with a 95% confidence interval of [0,17.48].

According to the result obtained in Fig. 5f, around two thirds are normal with respect to ED, and 36% have the ED with mild to moderate severity. There were no severe or extreme cases of ED found in the responded population.

#### Screening for OCD

The responses for the ASQ 12–13 were analyzed without encoding to screen for the OCD. According to the results, 34% of the students were found with the presence of OCD.

#### Screening for schizophrenia

An analysis after numerical encoding of the student’s responses for mini-FROGS questionnaire yields 29% of the students showing symptoms for schizophrenia, while 71% are normal with respect to schizophrenia. The mean score for the mini-FROGS questionnaire was 5.47, having a 95% confidence interval of [0, 12.44].

#### Screening for paranoia

The responses for PWQ-5 were numerically encoded, and analyzed. According to the analysis, 42% were screened as paranoia present, while 58% were screened as normal. The mean score for the PWQ-5 questionnaire was 4.03, having a 95% confidence interval of [0,12.43].

#### Screening for PTSD

The responses for ASQ 14–17 were numerically encoded, and analyzed. Only 3% of the students were screened as suffering from PTSD according to the analyzed results. Out of those who were screened positive, 33% (1) was reported due to ragging. But this evidence is not sufficient to claim whether all or most of the ragging cases directly affects PTSD or not. It only proves that there is a low possibility of causing PTSD due to ragging.

#### Screening for psychosis

The students’ responses for PQ were encoded numerically, and analyzed to obtain the psychosis classes as shown in Fig. 5g. The mean value for the score for PQ was 9.08, with a 95% confidence interval of [0,26.86].

According to the results depicted in Fig. 5g, 28% were screened positive for psychosis and out of those who were positive, none showed severe psychosis. Most of the students who were positive for psychosis had mild psychosis characteristics, and only 10% were screened with moderate psychosis symptoms.

### Overall analysis with psychiatric illnesses

**Figure 6:**
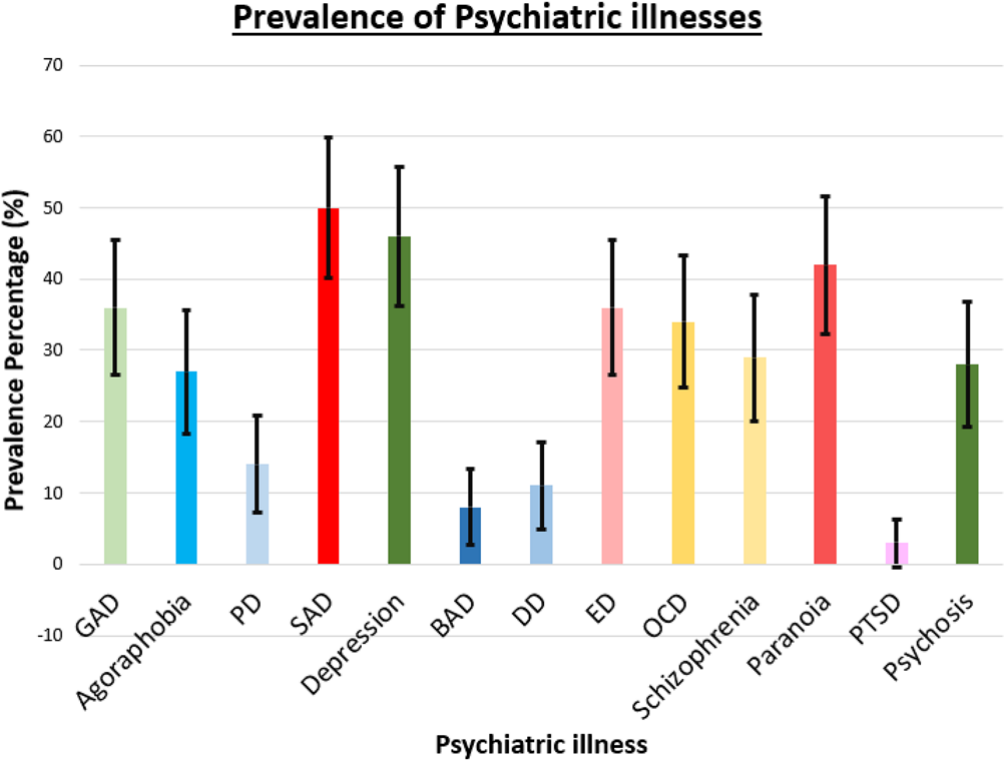
Prevalence rate of psychiatric illnesses among university students.

In this section, we convert all multi-class classifications of psychiatric illnesses into binary classes. The two classes are “Normal” and “Psychiatric illness present”, where psychiatric illness is one of the 13 illnesses analyzed in this research. Here, we put the class “Possible” also as “Normal”, and all other classes such as “Mild”, “Moderate”, “Severe” into “Psychiatric illness present class”. After the classification, we plot the percentage of “psychiatric illness present” class along with the 95% confidence interval as shown in the Fig. 6.

According to the result in Fig. 6, the descending order of prevalence percentage of psychiatric illnesses is SAD, depression, paranoia, ED, GAD, OCD, schizophrenia, psychosis, agoraphobia, PD, DD, BAD, and PTSD. The prevalence percentage of PTSD is low as 3%, and that of SAD is high as 50%. Another important thing to note here is that all psychiatric illnesses have a set of students screened positive. Therefore, the mean and the 95% confidence interval for prevalence of any psychiatric illness among students can be calculated using the result obtained in Fig. 6 as 28 and [0.00, 56.72] respectively.

We further summarize the prevalence of psychiatric illnesses with prevalence percentages, 95% confidence intervals, and Chi-Squared statistic calculated between the variables prevalence and gender, as seen in Table 2.

**Table 2:** Summary table showing the prevalence percentages with 95% confidence intervals, and Chi-Squared statistic between the prevalence and gender.

Psychiatric illness	Prevalence percentage	95% confidence interval	Chi-squared statistic
GAD	36	[26.59, 45.41]	0.8823
Agoraphobia	27	[18.30, 35.70]	0.2726
PD	14	[7.19, 20.80]	0.0542
SAD	50	[40.2, 59.8]	3.6635
Depression	46	[36.23, 55.77]	0.0059
BAD	8	[2.68, 13.32]	0.2517
DD	11	[4.87, 17.13]	0.1834
ED	36	[26.59, 45.41]	0.1520
OCD	34	[24.72, 43.28]	0.0098
Schizophrenia	29	[20.11, 37.89]	4.3108
Paranoia	42	[32.33, 51.67]	1.8306
PTSD	3	[0.00, 6.34]	1.5233
Psychosis	28	[19.20, 36.80]	0.0129

**Figure 7:**
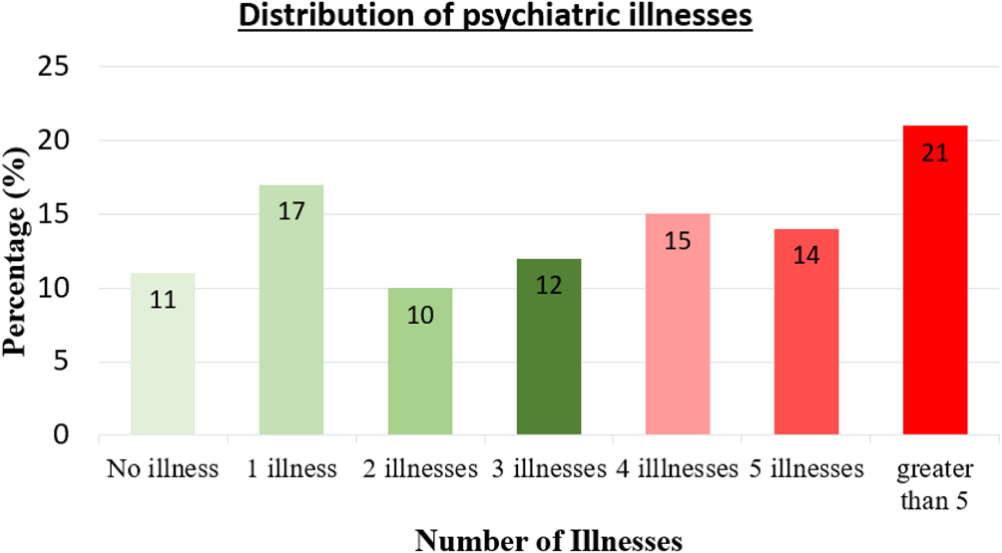
Psychiatric illness distribution among students.

Now let us analyze the distribution of psychiatric illnesses. Here, we check the students without any psychiatric illness against who have only 1, only 2, only 3, only 4, only 5, and greater than 5 psychiatric illnesses, as shown in Fig. 7.

As evident from the column chart given in Fig. 7, 89% of the students have 1 or more psychiatric illnesses. Further, the students with 1 illness is only 17%. So, the students with multiple psychiatric illnesses are high as 72%, which can be considered as a danger sign. This result proves that majority of the students need to seek psychiatric assistance from trained professionals. On the other hand, only 8% have visited a psychiatrist and sought medical advice. Therefore, there is a huge gap among psychiatrically ill students those who have seek medical advice, and those who haven’t. For instance, as it was shown in the section on already diagnosed diseases; they have not been diagnosed for agoraphobia, BAD, DD, schizophrenia, and paranoia. However, as proved in this section, there are indeed students who have been screened positive for these undiagnosed diseases as well in large numbers.

### Statistical analysis

In this section, we statistically analyze the correlation between the prevalence of psychiatric illnesses, and identify the risk factors contributing for the prevalence of each psychiatric illness. For Binary Logistic Regression (BLR), we first encode the population characteristics, academic stressors, and the environmental stressors as the predictors, and the presence/absence of a particular illness as the dependent variable.

#### Correlation between the prevalence of psychiatric illnesses

In order to understand the correlation between the illnesses, we find out the Pearson correlation coefficient for the presence or absence of an illness between the psychiatric illnesses. The calculated coefficients are as shown in Table 3.

**Table 3:** Pearson correlation coefficient between the occurrence of psychiatric illnesses among students.

	GAD	Agor.	PD	SAD	Depr.	BAD	DD	ED	OCD	schi.	paran.	PTSD	Psych.
GAD	1.00	0.25	0.12	0.25	**0.39**	0.01	0.00	0.05	0.08	0.26	0.25	0.11	**0.37**
Agor.	0.25	1.00	0.27	0.20	**0.48**	− 0.01	0.29	0.15	0.13	0.16	0.21	0.29	**0.32**
PD	0.12	0.27	1.00	0.17	0.09	0.09	0.13	0.06	0.20	0.12	0.12	0.10	− 0.06
SAD	0.25	0.20	0.17	1.00	**0.32**	0.00	0.16	0.08	0.00	0.07	0.20	− 0.06	0.22
Depr.	**0.39**	**0.48**	0.09	**0.32**	1.00	0.25	0.12	0.14	0.14	0.07	**0.35**	0.19	**0.36**
BAD	0.01	− 0.01	0.09	0.00	0.25	1.00	0.01	0.01	0.02	− 0.03	0.12	0.16	− 0.02
DD	0.00	0.29	0.13	0.16	0.12	0.01	1.00	0.20	0.29	0.06	**0.35**	** 0.31**	0.42
ED	0.05	0.15	0.06	0.08	0.14	0.01	0.20	1.00	0.12	**0.30**	0.29	− 0.01	0.27
OCD	0.08	0.13	0.20	0.00	0.14	0.02	0.29	0.12	1.00	0.05	**0.33**	0.12	0.16
schi.	0.26	0.16	0.12	0.07	0.07	− 0.03	0.06	**0.30**	0.05	1.00	0.13	− 0.11	0.19
paran.	0.25	0.21	0.12	0.20	**0.35**	0.12	**0.35**	0.29	**0.33**	0.13	1.00	0.09	**0.42**
PTSD	0.11	0.29	0.10	− 0.06	0.19	0.16	**0.31**	− 0.01	0.12	− 0.11	0.09	1.00	0.28
Psych.	**0.37**	**0.32**	− 0.06	0.22	**0.36**	− 0.02	**0.42**	0.27	0.16	0.19	**0.42**	0.28	1.00

As evident from the correlation coefficients in Table 3, there was no pair of diseases which showed strong correlation between each other. However, as observed from the bold coefficients in Table 3, medium correlation had existed between the following pairs of illnesses.

- GAD and depression.
- GAD and psychosis.
- Agoraphobia and depression.
- SAD and depression.
- Depression and paranoia.
- Depression and psychosis.
- DD and paranoia.
- DD and PTSD.
- DD and psychosis.
- ED and schizophrenia.
- OCD and paranoia.
- Paranoia and psychosis.

All the negative correlation coefficients are small (less than 0.3); hence even though negative correlation exists, such effects are minimum. Therefore, the possibility of coexisting psychological illnesses between above pairs has been medium.

#### Factors contributing for GAD

Binary logistic regression coefficients, odds ratio, *p*-value, and 95 % confidence interval for each of the predictors of GAD are given in Table 4. Acronyms “AS” stands for Academic Stressor and “ES” stands for Environmental Stressor in Table 4, and they are listed in Sect. 2.3.

**Table 4:** Binary logistic regression coefficient, odds ratio, *p*-value, and 95% confidence interval table for predicting GAD.

Predictor	Coefficient	Odds ratio	*p*-value	95% CI	Predictor	Coefficient	Odds ratio	*p*-value	95% CI
Gender	0.34	1.41	0.074	[0.19, 10.64]	Age	− 0.49	0.61	0.037	[0.20, 1.81]
Civil status	− 20.44	0.00	0.999	[0,inf]	Ethnicity	− 0.39	0.67	0.078	[0.04,10.71]
Religion	0.8	2.22	0.027	[0.54,9.08]	Academic year	0.03	1.03	0.097	[0.23,4.60]
Current OGPA	0.73	2.08	0.042	[0.35,12.26]	Degree class	0.21	1.24	0.069	[0.43,3.51]
Income	− 0.03	0.97	0.084	[0.68,1.35]	AS1	− 0.06	0.95	0.092	[0.33,2.68]
AS2	− 0.27	0.76	0.062	[0.25,2.28]	AS3	− 0.06	0.94	0.090	[0.37,2.39]
AS4	0.30	1.35	0.061	[0.43,4.26]	AS5	− 0.93	0.39	0.013	[0.12, 1.32]
**AS6**	0.82	2.26	0.011	[0.82,6.22]	**AS7**	0.26	1.30	0.068	[0.37,4.55]
AS8	− 2.28	0.10	0.000	[0.02,0.46]	**AS9**	0.18	1.20	0.074	[0.40,3.51]
**AS10**	0.85	2.33	0.009	[0.86,6.33]	**AS11**	0.99	2.68	0.011	[0.80,8.96]
AS12	− 0.13	0.87	0.085	[0.22,3.41]	**AS13**	0.49	1.63	0.030	[0.64,4.14]
AS14	− 0.14	0.87	0.072	[0.39,1.91]	**ES1**	0.29	1.33	0.055	[0.52,3.44]
**ES2**	0.82	2.28	0.007	[0.95,5.46]	**ES3**	0.36	1.43	0.041	[0.62,3.30]
**ES4**	0.75	2.13	0.003	[1.08,4.19]	ES5	− 0.91	0.40	0.002	[0.18,0.89]
**ES6**	0.16	1.18	0.066	[0.58,2.40]	**ES7**	0.93	2.52	0.003	[1.09,5.82]
ES8	− 0.63	0.53	0.015	[0.23,1.27]	ES9	− 0.49	0.61	0.048	[0.16,2.38]
ES10	− 0.72	0.49	0.006	[0.23,1.04]	**ES11**	0.46	1.59	0.025	[0.73,3.48]

As seen in Table 4, BLR yields a coefficient of 0.34 and an odds ratio of 1.40 for the gender, concluding that there is 40% higher chance for finding GAD in males than females. Probability of finding GAD seems to reduce with the increment of age, as coefficient being negative (− 0.49) and having odds ratio of 0.61. However, with academic year, the probability of finding GAD increases by 3%. A correlation between the prevalence of GAD and the civil status cannot be obtained from BLR due to the high statistical non-significance suggested by *p*-value of 0.999 for the variable civil status. As odds ratio for ethnicity is 0.67, there is a low possibility of occurring GAD when the race is not Sinhalese. A positive coefficient (0.8) in religion with an odds ratio of 2.22 indicates that when the student is a non-Buddhist; the tendency for having GAD increases. OGPA and degree class also contribute positively for having GAD among students, suggested by positive coefficients and large odds ratios, as seen in Table 4. With the increment of family income class, the tendency to find GAD decreases, as suggested by a negative coefficient of − 0.03. As shown in bold fonts in Table 4, the academic stressors 6, 7, 9, 10, 11, and 13 and environmental stressors 1, 2, 3, 4, 6, 7, and 11 have contributed positively for the prevalence of GAD in students, and thus can be considered as risk factors for GAD.

#### Factors contributing for agoraphobia

Binary logistic regression coefficients, odds ratio, *p*-value, and 95 % confidence interval for each of the predictors of agoraphobia are given in Table 5.

**Table 5:** Binary logistic regression coefficient, odds ratio, *p*-value, and 95% confidence interval table for predicting agoraphobia.

Predictor	Coefficient	Odds ratio	*p*-value	95% CI	Predictor	Coefficient	Odds ratio	*p*-value	95% CI
Gender	4.21	67.63	0.005	[0.97,4739]	Age	− 2.08	0.12	0.016	[0.01,2.32]
Civil status	36.17	5.13E15	0.360	[0,2.0E49]	Ethnicity	12.88	3.93E05	0.001	[33.06, 4.68E09]
Religion	− 14.64	0.00	0.000	[0.00,0.01]	Academic year	5.06	157.28	0.001	[3.16,7837.49]
Current OGPA	− 1.92	0.15	0.018	[0.01,2.46]	Degree class	− 2.77	0.06	0.002	[0.01,0.69]
Income	2.52	12.44	0.000	[2.31,67.08]	AS1	− 3.58	0.03	0.001	[0.00,0.39]
AS2	− 4.71	0.01	0.001	[0.00,0.25]	**AS3**	1.92	6.82	0.008	[0.77,60.17]
**AS4**	9.11	9.02E03	0.000	[19.03, 4.27E06]	AS5	− 3.09	0.05	0.006	[0.00,1.08]
**AS6**	6.25	518.02	0.000	[16.37,16389]	AS7	− 2.7	0.07	0.009	[0.00,1.51]
**AS8**	0.53	1.70	0.073	[0.08,34.57]	**AS9**	1.83	6.22	0.011	[0.66,58.62]
**AS10**	2.50	12.23	0.008	[0.73,205.51]	AS11	− 1.93	0.14	0.013	[0.01,1.78]
AS12	− 10.5	0.00	0.000	[0.00,0.01]	**AS13**	10.06	2.34E04	0.000	[27.43, 1.99E07]
**AS14**	7.33	1.52E03	0.000	[13.72,1.68E05]	ES1	− 1.00	0.37	0.027	[0.06,2.20]
**ES2**	7.97	2.91E03	0.000	[22.36, 3.77E05]	ES3	− 4.58	0.01	0.002	[0.00,0.53]
**ES4**	2.07	7.95	0.001	[1.78,35.45]	**ES5**	3.49	32.93	0.002	[1.70,637.46]
ES6	− 6.49	0.00	0.000	[0.00,0.08]	ES7	− 0.53	0.59	0.053	[0.11,3.05]
**ES8**	4.8	121.97	0.001	[2.60,5724.33]	ES9	− 8.67	0.00	0.001	[0.00,0.08]
**ES10**	0.07	1.07	0.093	[0.21,5.43]	ES11	− 2.46	0.09	0.016	[0.00,2.65]

As seen in Table 5, BLR yields a coefficient of 4.21 and an odds ratio of 67.63 for the gender, concluding that there is a very higher chance for finding agoraphobia in males than females. Probability of finding agoraphobia seems to reduce with the increment of age, as coefficient being negative (− 2.08) and having odds ratio of 0.12. However, with academic year, the probability of finding agoraphobia increases very significantly, as proved by the odds ratio of 157.28. A correlation between the prevalence of agoraphobia and the civil status cannot be obtained from BLR, due to the high statistical non-significance suggested by *p*-value of 0.36 for the variable civil status. As odds ratio for ethnicity is 3.93E05, there is very high possibility of occurring agoraphobia when the race is not Sinhalese. A negative coefficient (− 14.64) in religion with an odds ratio of 0.00 indicates that when the student is a Buddhist, the tendency for having agoraphobia is very high. Current OGPA and degree class contribute negatively for having agoraphobia among students, suggested by negative coefficients and small odds ratios, as seen in Table 5. The presence of agoraphobia tends to increase with the increment of the income class, as suggested by positive coefficient of 2.52. As shown in bold fonts in Table 5, the academic stressors 3, 4, 8, 9, 10, 13, and 14 and environmental stressors 2, 4, 5, 8, and 10 have contributed positively for the prevalence of agoraphobia in students, and thus can be considered as risk factors for agoraphobia.

#### Factors contributing for PD

Binary logistic regression coefficients, odds ratio, *p*-value, and 95 % confidence interval for each of the predictors of PD are given in Table 6.

**Table 6:** Binary logistic regression coefficient, odds ratio, *p*-value, and 95% confidence interval table for predicting PD.

Predictor	Coefficient	Odds ratio	*p*-value	95% CI	Predictor	Coefficient	Odds ratio	*p*-value	95% CI
Gender	− 6.5	0.00	0.089	[1.57E-40, 1.44E34]	Age	− 0.49	0.61	0.072	[3.03E-45, 2.70E30]
Civil status	− 20.74	0.00	0.940	[1.3E-230,7.4E211]	Ethnicity	23.78	2.12E10	0.084	[4.3E-88,1E108]
Religion	2.83	16.91	0.094	[1.5E-30,2.49E32]	Academic year	0.18	1.20	1.000	[1.09E-25,1.32E25]
Current OGPA	23.84	2.26E10	0.061	[1.39E-28,3.67E48]	Degree class	− 35.63	0	0.042	[2.04E-52,5.5E20]
Income	3.33	28.04	0.041	[0.014,55386]	**AS1**	8.93	7.58E03	0.037	[6.29E-5,9.14E11]
AS2	− 25.08	0.00	0.047	[7.80E-40,2.12E17]	**AS3**	5.47	237.41	0.083	[9.42E-20,5.98E23]
**AS4**	23.57	1.73E10	0.055	[1.31E-22,2.28E42]	AS5	− 13.94	0.00	0.049	[2.2E-23,3.52E10]
**AS6**	6.06	427.66	0.085	[3.75E-24,4.88E28]	**AS7**	9.63	1.52E04	0.068	[1.1E-15,2.11E23]
AS8	− 15.81	0.00	0.086	[4.03E-80,4.6E65]	**AS9**	3.26	26.10	0.087	[4.45E-16,1.53E18]
**AS10**	29.09	4.28E12	0.031	[1.07E-11,1.71E36]	AS11	− 13.38	0.00	0.068	[9.25E-33,2.58E20]
**AS12**	25.9	1.77E11	0.071	[3.84E-47,8.17E68]	**AS13**	0.29	1.34	0.099	[1.1E-28,1.63E28]
AS14	− 14.39	0.00	0.041	[2.29E-21,1.39E08]	**ES1**	3.23	25.33	0.087	[4.89E-15,1.31E17]
ES2	− 7.04	0.00	0.064	[4.09E-16,1.89E09]	**ES3**	19.52	2.99E8	0.065	[4.82E-27,1.86E43]
**ES4**	4.68	107.55	0.087	[1.16E-21,9.99E24]	**ES5**	14.25	1.55E06	0.065	[1.59E-20, 1.51E32]
ES6	− 20.09	0.00	0.023	[2.95E-23,1.2E05]	**ES7**	5.07	159.23	0.070	[4.22E-9,6.01E12]
**ES8**	1.83	6.25	0.088	[3.44E-10,1.14E11]	**ES9**	14.67	2.36E06	0.065	[9.64E-21,5.79E32]
**ES10**	0.95	2.6	0.097	[1.54E-19,4.38E19]	**ES11**	3.80	44.82	0.091	[3.17E-25,6.33E27]

As seen in Table 6, BLR yields a coefficient of -6.5 and an odds ratio of 0.00 for the gender, concluding that there is a very higher chance for finding PD in females than males. Probability of finding PD seems to reduce with the increment of age, as coefficient being negative (-0.49) and having odds ratio of 0.61. However, with academic year, the probability of finding PD increases by 20%. A correlation between the prevalence of PD and the civil status cannot be obtained from BLR, due to the high statistical non-significance suggested by *p*-value of 0.94 for the variable civil status. As odds ratio for ethnicity is 2.12E10, there is a very high possibility of occurring PD when the race is not Sinhalese. A positive coefficient (2.83) in religion with an odds ratio of 16.91 indicates that when the student is a non-Buddhist, the tendency for having PD increases drastically. Current OGPA increment tends to increase the chance of finding students with PD, as proved by positive coefficient of 23.84. But, the degree class contributes negatively for having PD among students, suggested by negative coefficients and zero odds ratio as seen in Table 6. The increment of the family income class increases the probability of finding a student with PD, as suggested by positive coefficient of 3.33. As shown in bold fonts in Table 6, the academic stressors 1, 3, 4, 6, 7, 9, 10, 12, and 13 and environmental stressors 1, 3, 4, 5, 7, 8, 9, 10, and 11 have contributed positively for the prevalence of PD in students, and thus can be considered as risk factors for PD.

#### Factors contributing for SAD

Binary logistic regression coefficients, odds ratio, *p*-value, and 95 % confidence interval for each of the predictors of SAD are given in Table 7.

**Table 7:** Binary logistic regression coefficient, odds ratio, *p*-value, and 95% confidence interval table for predicting SAD.

Predictor	Coefficient	Odds ratio	*p*-value	95% CI	Predictor	Coefficient	Odds ratio	*p*-value	95% CI
Gender	− 1.43	0.24	0.008	[0.05,1.17]	Age	− 0.89	0.41	0.008	[0.15,1.13]
Civil status	− 21.99	0.00	1.000	[0,inf]	Ethnicity	− 4.25	0.01	0.011	[0.00,2.73]
Religion	2.55	12.86	0.001	[1.76,94.03]	Academic year	1.34	3.84	0.006	[0.94,15.69]
Current OGPA	0.16	1.18	0.079	[0.36,3.89]	Degree class	− 0.08	0.93	0.086	[0.39,2.18]
Income	− 0.26	0.77	0.007	[0.58,1.02]	AS1	− 0.25	0.78	0.056	[0.34,1.78]
**AS2**	0.41	1.51	0.040	[0.58,3.90]	**AS3**	0.11	1.12	0.075	[0.56,2.25]
**AS4**	0.03	1.03	0.095	[0.43,2.47]	AS5	− 0.54	0.58	0.027	[0.22,1.53]
**AS6**	0.09	1.10	0.080	[0.54,2.24]	**AS7**	0.7	2.01	0.018	[0.73,5.55]
AS8	− 0.84	0.43	0.090	[0.17,1.13]	**AS9**	0.36	1.43	0.041	[0.61,3.34]
**AS10**	0.30	1.36	0.041	[0.66,2.78]	AS11	− 0.49	0.61	0.029	[0.25,1.52]
**AS12**	0.23	1.26	0.070	[0.39,4.03]	AS13	− 0.14	0.87	0.074	[0.38,1.97]
**AS14**	0.31	1.36	0.038	[0.68,2.72]	**ES1**	0.01	1.01	0.098	[0.49,2.08]
**ES2**	0.62	1.86	0.009	[0.91,3.82]	**ES3**	0.63	1.88	0.005	[0.99,3.56]
**ES4**	0.19	1.21	0.046	[0.74,1.98]	ES5	− 0.37	0.69	0.025	[0.37,1.29]
**ES6**	0.04	1.04	0.090	[0.56,1.94]	**ES7**	0.03	1.03	0.092	[0.56,1.90]
ES8	− 0.68	0.51	0.006	[0.25,1.04]	**ES9**	0.92	2.51	0.006	[0.95,6.65]
ES10	− 0.04	0.96	0.088	[0.58,1.60]	**ES11**	0.21	1.24	0.047	[0.69,2.21]

As seen in Table 7, BLR yields a coefficient of − 1.43 and an odds ratio of 0.24 for the gender, concluding that there is 76% higher chance for finding SAD in females than males. Probability of finding SAD seems to reduce with the increment of age, as coefficient being negative (− 0.89) and having odds ratio of 0.41. However, with academic year, the probability of finding SAD increases by a very large margin (284% increment per academic year). A correlation between the prevalence of SAD and the civil status cannot be obtained from BLR, due to the high statistical non-significance suggested by *p*-value of 1.0 for the variable civil status. As odds ratio for ethnicity is 0.01, there is a low possibility of occurring SAD when the race is not Sinhalese. A positive coefficient (2.55) in religion with an odds ratio of 12.86 indicates that when the student is a non-Buddhist, the tendency for having SAD dramatically increases. Due to positive coefficient (0.16) and odds ratio (1.18), OGPA also contribute positively for having SAD among students. On the other hand, degree class has a small negative coefficient (− 0.08) indicating SAD decreases slightly with increment of the degree class, as seen in Table 7. With the increment of income, SAD tends to decrease, as suggested by a negative coefficient of − 0.26. As shown in bold fonts in Table 7, the academic stressors 2, 3, 4, 6, 7, 9, 10, 12, and 14 and environmental stressors 1, 2, 3, 4, 6, 7, 9, and 11 have contributed positively for the prevalence of SAD in students, and thus can be considered as risk factors for SAD.

#### Factors contributing for depression

Binary logistic regression coefficients, odds ratio, *p*-value, and 95 % confidence interval for each of the predictors of depression are given in Table 8.

**Table 8:** Binary logistic regression coefficient, odds ratio, *p*-value, and 95% confidence interval table for predicting depression.

Predictor	Coefficient	Odds ratio	*p*-value	95% CI	Predictor	Coefficient	Odds ratio	*p*-value	95% CI
Gender	0.97	2.63	0.018	[0.63,10.94]	Age	0.82	2.28	0.008	[0.92,5.63]
Civil status	− 21.47	0.00	1.000	[0,inf]	Ethnicity	− 1.83	0.16	0.010	[0.02,1.46]
Religion	1.10	3.02	0.006	[0.94,9.68]	Academic year	− 0.35	0.7	0.051	[0.25,2.00]
Current OGPA	− 0.39	0.68	0.045	[0.25,1.87]	Degree class	− 0.17	0.85	0.067	[0.39,1.82]
Income	0.14	1.15	0.024	[0.91,1.46]	AS1	− 0.68	0.51	0.011	[0.22,1.17]
AS2	− 0.28	0.75	0.053	[0.31,1.81]	**AS3**	0.31	1.37	0.035	[0.71,2.63]
**AS4**	0.29	1.34	0.048	[0.60,2.99]	AS5	− 0.28	0.76	0.051	[0.33,1.73]
**AS6**	0.23	1.26	0.046	[0.69,2.29]	**AS7**	0.15	1.16	0.076	[0.44,3.08]
AS8	− 0.99	0.37	0.003	[0.15,0.91]	**AS9**	0.63	1.89	0.012	[0.85,4.20]
**AS10**	0.16	1.17	0.064	[0.60,2.28]	AS11	− 0.24	0.79	0.055	[0.36,1.72]
AS12	− 0.53	0.59	0.034	[0.20,1.75]	**AS13**	0.93	2.53	0.003	[1.07,5.97]
AS14	− 0.05	0.95	0.089	[0.51,1.80]	ES1	− 0.31	0.73	0.037	[0.38,1.44]
**ES2**	1.03	2.81	0.001	[1.26,6.24]	**ES3**	0.06	1.07	0.083	[0.60,1.88]
**ES4**	0.32	1.37	0.016	[0.88,2.13]	ES5	− 0.36	0.70	0.017	[0.41,1.18]
ES6	− 0.14	0.87	0.064	[0.48,1.57]	**ES7**	0.32	1.38	0.022	[0.82,2.30]
ES8	− 0.4	0.67	0.022	[0.35,1.27]	ES9	− 0.03	0.97	0.095	[0.41,2.31]
ES10	− 0.41	0.66	0.010	[0.41,1.08]	ES11	− 0.01	0.99	0.099	[0.55,1.79]

As seen in Table 8, BLR yields a coefficient of 0.97 and an odds ratio of 2.63 for the gender, concluding that there is 163% higher chance for finding depression in males than females. Probability of finding depression seems to increase with the increment of age, as coefficient being positive (0.82) and having odds ratio of 2.28. However, with academic year, the probability of finding depression decreases by 30%. A correlation between the prevalence of depression and the civil status cannot be obtained from BLR, due to the high statistical non-significance suggested by *p*-value of 1.0 for the variable civil status. As odds ratio for ethnicity is -1.83, there is less possibility of occurring depression when the race is not Sinhalese. A positive coefficient (1.10) in religion with an odds ratio of 3.02 indicates that when the student is a non-Buddhist, the tendency for having depression increases. OGPA and degree class contribute negatively for having depression among students, suggested by negative coefficients and less than 1 odds ratios, as seen in Table 8. With the increment of family income, there is a high chance for the increment of depression, as suggested by a coefficient of 0.14 and odds ratio of 1.15. As shown in bold fonts in Table 8, the academic stressors 3, 4, 6, 7, 9, 10, and 13 and environmental stressors 2, 3, 4, and 7 have contributed positively for the prevalence of depression in students, and thus can be considered as risk factors for depression.

#### Factors contributing for BAD

Binary logistic regression coefficients, odds ratio, *p*-value, and 95 % confidence interval for each of the predictors of BAD are given in Table 9.

**Table 9:** Binary logistic regression coefficient, odds ratio, *p*-value, and 95% confidence interval table for predicting BAD.

Predictor	Coefficient	Odds ratio	*p*-value	95% CI	Predictor	Coefficient	Odds ratio	*p*-value	95% CI
Gender	37.57	2.07E16	0.070	[0.04,2.07E29]	Age	− 6	0.00	0.069	[0.00,1.99]
Civil status	248.72	1.04E108	0.990	[0,inf]	Ethnicity	− 28.77	0.00	0.013	[0.00,1.67]
Religion	14.58	2.14E06	0.031	[0.45,3.37E11]	Academic year	8.38	4.38E03	0.055	[0.57,6.86E09]
Current OGPA	8.54	5.11E03	0.047	[0.39,6.37E10]	Degree class	− 9.6	0.0	0.081	[0.00,5.34]
Income	14.45	1.88E06	0.001	[0.69,3.54E13]	AS1	− 32.72	0.00	0.043	[0.00,3.54]
AS2	− 15.97	0.00	0.082	[0.00,3.92]	AS3	− 22.71	0.00	0.071	[0.00,3.17]
**AS4**	6.15	469	0.062	[0.97,5432]	**AS5**	0.94	2.56	0.027	[0.53,17.87]
**AS6**	16.5	1.46E07	0.072	[0.06,1.06E18]	AS7	− 21.71	0.00	0.065	[0.00,0.84]
**AS8**	6.8	902	0.059	[0.47,8563]	**AS9**	12.46	2.58E05	0.079	[0.06,8.43E14]
**AS10**	0.13	1.14	0.052	[0.04,17.32]	**AS11**	8.65	5.70E03	0.007	[0.14,2.87E10]
AS12	− 5.83	0.00	0.086	[0.00,1.62]	AS13	− 4.18	0.02	0.074	[0.005,2.27]
**AS14**	8.19	3.60E03	0.006	[0.32,5.27E11]	**ES1**	0.8	2.23	0.001	[1.16,19.36]
**ES2**	5.04	155	0.010	[0.56,1953]	**ES3**	11.73	1.24E05	0.042	[0.03,3.27E14]
ES4	− 14.79	0.00	0.021	[0.00,1.21]	**ES5**	0.42	1.52	0.012	[0.13,18.64]
ES6	− 4.30	0.01	0.008	[0.00,1.51]	ES7	− 6.63	0.00	0.042	[0.00,2.34]
**ES8**	2.52	12.4	0.036	[0.36,138.7]	ES9	− 30.02	0.00	0.002	[0.00,0.97]
**ES10**	5.41	224.02	0.051	[0.52,5223]	**ES11**	3.54	34.57	0.080	[0.29,789.2]

As seen in Table 9, BLR yields a coefficient of 37.57 and an odds ratio of 2.07E16 for the gender, concluding that there is very higher chance for finding BAD in males than females. Probability of finding BAD seems to reduce with the increment of age, as coefficient being negative (− 6) and having odds ratio of 0.00. However, with academic year, the probability of finding BAD increases by very large margin due to coefficient being 8.38 with odds ratio 438. A correlation between the prevalence of BAD and the civil status cannot be obtained from BLR, due to the high statistical non-significance suggested by *p*-value of 0.990 for the variable civil status. As odds ratio for ethnicity is − 28.77, there is a high possibility of occurring BAD when the race is Sinhalese. A positive coefficient (14.58) in religion with an odds ratio of 2.14E06 indicates that when the student is a non-Buddhist, the tendency for having BAD increases. OGPA also contributes positively for having BAD among students, suggested by positive coefficients and large odds ratios, as seen in Table 9. With the increment of degree class, BAD is less likely to be found due to the negative coefficient and zero odds ratio, suggesting that BAD is almost found in general degree holders. With increment of income, there is a high chance of finding BAD due to positive coefficient of 14.45 and odds ratio of 1.88E06. As shown in bold fonts in Table 9, the academic stressors 4, 5, 6, 8, 9, 10, 11, and 14 and environmental stressors 1, 2, 3, 5, 8, 10, and 11 have contributed positively for the prevalence of BAD in students, and thus can be considered as risk factors for BAD.

#### Factors contributing for DD

Binary logistic regression coefficients, odds ratio, *p*-value, and 95 % confidence interval for each of the predictors of DD are given in Table 10.

**Table 10:** Binary logistic regression coefficient, odds ratio, *p*-value, and 95% confidence interval table for predicting DD.

Predictor	Coefficient	Odds ratio	*p*-value	95% CI	Predictor	Coefficient	Odds ratio	*p*-value	95% CI
Gender	32.44	1.2E14	0.018	[0.26, 3.8E25]	Age	− 15.43	2.0E-07	0.092	[0.00,0.01]
Civil status	260.48	1.3E113	1.000	[0,inf]	Ethnicity	43.23	5.9E+18	0.071	[0.13,2.23E31]
Religion	− 11.03	1.6E-05	0.004	[0.00,0.04]	Academic year	61.7	6.2E26	0.047	[0.51, 7.25E41]
Current OGPA	− 46.41	7.0E-21	0.018	[0.00,1.51E-5]	Degree class	16.31	1.2E07	0.070	[0.17,3.23E17]
Income	7.57	1.9E03	0.028	[0.34,1.91E07]	AS1	− 8.3	2.5E-04	0.088	[0.00,0.09]
AS2	− 35.88	2.6E-16	0.048	[0.00,6.31E-4]	**AS3**	5.92	370	0.007	[0.91, 2105]
**AS4**	37.1	1.3E16	0.019	[0.74, 4.56E29]	**AS5**	10.06	2.3E04	0.078	[0.52,8.23E09]
**AS6**	19.63	3.3E08	0.080	[0.16,5.92E17]	AS7	− 8.6	1.8E-04	0.084	[0.00,0.14]
**AS8**	12.93	4.1E05	0.049	[0.34,3.21E12]	**AS9**	32.29	1.1E14	0.024	[0.14,6.78E25]
AS10	− 11.65	8.7E-06	0.061	[0.00,0.06]	AS11	− 13.39	1.5E-06	0.087	[0.00,0.11]
AS12	− 39.79	5.2E-18	0.021	[0.00,9.1E-05]	**AS13**	30.35	1.5E13	0.078	[0.39,4.2E27]
**AS14**	24.66	5.1E10	0.058	[0.31,6.1E17]	ES1	0.00	1.00	0.013	[0.31,15.21]
**ES2**	0.95	2.6	0.005	[0.41,31.24]	**ES3**	8.85	7000	0.034	[0.51,1.23E6]
**ES4**	2.43	11	0.053	[0.15,192.3]	ES5	− 24.11	3.4E-11	0.054	[0.00,6.2E-3]
ES6	− 18.59	8.5E-09	0.025	[0.00,7.5E-4]	ES7	− 3.22	0.04	0.011	[0.00,0.91]
**ES8**	21.04	1.4E09	0.058	[0.23,4.32E17]	ES9	− 22.48	1.7E-10	0.002	[0.00,7.3E-3]
**ES10**	9.41	1.2E04	0.020	[0.41,6.35E10]	ES11	− 21.80	3.4E-10	0.015	[0.00,5.6E-4]

As seen in Table 10, BLR yields a coefficient of 32.44 and an odds ratio of 1.2E14 for the gender, concluding that there is very higher chance for finding DD in males than females. Probability of finding DD seems to reduce drastically with the increment of age, as coefficient being negative (-15.43) and having odds ratio of 2.0E-07. However, with academic year, the probability of finding DD increases by a very large margin, as suggested by high odds ratio of 6.2E26. A correlation between the prevalence of DD and the civil status cannot be obtained from BLR, due to the high statistical non-significance suggested by *p*-value of 1.0 for the variable civil status. As odds ratio for ethnicity is 5.9E18, there is high possibility of occurring DD when the race is not Sinhalese. A negative coefficient (-11.03) in religion with an odds ratio of 1.6E-05 indicates that when the student is a non-Buddhist; the tendency for having DD decreases drastically. OGPA seems to contribute negatively for having DD among students, suggested by negative coefficient and large odds ratio, as seen in Table 10. Degree class has a positive relationship for the DD, as suggested by the positive coefficient of 16.31. Increment of family income also contributes positively for the DD, since the corresponding coefficient is 7.57. As shown in bold fonts in Table 10, the academic stressors 3, 4, 5, 6, 8, 9, 13, and 14 and environmental stressors 2, 3, 4, 8, and 10 have contributed positively for the prevalence of DD in students, and thus can be considered as risk factors for DD.

#### Factors contributing for ED

Binary logistic regression coefficients, odds ratio, *p*-value, and 95 % confidence interval for each of the predictors of ED are given in Table 11.

**Table 11:** Binary logistic regression coefficient, odds ratio, *p*-value, and 95% confidence interval table for predicting ED.

Predictor	Coefficient	Odds ratio	*p*-value	95% CI	Predictor	Coefficient	Odds ratio	*p*-value	95% CI
Gender	0.70	2.02	0.028	[0.56,7.25]	Age	0.03	1.03	0.092	[0.52,2.06]
Civil status	− 19.54	0.00	1.000	[0,inf]	Ethnicity	− 0.18	0.84	0.081	[0.20,3.57]
Religion	0.71	2.03	0.014	[0.80,5.16]	Academic year	0.36	1.43	0.047	[0.54,3.82]
Current OGPA	− 0.55	0.57	0.028	[0.21,1.57]	Degree class	0.08	1.09	0.080	[0.57,2.07]
Income	0.10	1.10	0.038	[0.89,1.37]	AS1	− 0.01	0.99	0.098	[0.51,1.94]
**AS2**	0.23	1.26	0.058	[0.55,2.89]	**AS3**	0.41	1.50	0.017	[0.84,2.68]
AS4	− 0.41	0.66	0.029	[0.31,1.41]	**AS5**	0.05	1.06	0.088	[0.51,2.20]
AS6	− 0.03	0.97	0.090	[0.55,1.69]	AS7	− 0.03	0.97	0.094	[0.42,2.23]
**AS8**	0.2	1.22	0.059	[0.59,2.53]	**AS9**	0.36	1.44	0.034	[0.68,3.04]
**AS10**	0.11	1.11	0.071	[0.63,1.98]	**AS11**	0.02	1.02	0.097	[0.44,2.32]
AS12	− 0.52	0.60	0.031	[0.22,1.61]	AS13	− 0.06	0.94	0.088	[0.44,2.00]
**AS14**	0.11	1.12	0.068	[0.66,1.91]	**ES1**	0.47	1.59	0.013	[0.88,2.90]
**ES2**	0.45	1.57	0.015	[0.84,2.93]	ES3	− 0.22	0.8	0.044	[0.46,1.40]
ES4	− 0.09	0.91	0.063	[0.62,1.34]	**ES5**	0.11	1.12	0.064	[0.70,1.77]
**ES6**	0.24	1.27	0.035	[0.77,2.10]	**ES7**	0.41	1.51	0.011	[0.92,2.48]
**ES8**	0.12	1.13	0.068	[0.63,2.05]	ES9	− 0.97	0.38	0.002	[0.17,0.83]
ES10	− 0.23	0.80	0.030	[0.52,1.23]	ES11	− 0.44	0.64	0.013	[0.36,1.15]

As seen in Table 11, BLR yields a coefficient of 0.70 and an odds ratio of 2.02 for the gender, concluding that there is 102% higher chance for finding ED in males than females. Probability of finding ED seems to slightly (by 3%) increase with the increment of age, as coefficient being positive (0.03) and having odds ratio of 1.03. This is true for the academic year which the probability of finding ED increases by 43%. A correlation between the prevalence of ED and the civil status cannot be obtained from BLR, due to the high statistical non-significance suggested by *p*-value of 1.0 for the variable civil status. As the odds ratio for ethnicity is 0.84, there is a low possibility of occurring ED when the race is not Sinhalese. A positive coefficient (0.71) in religion with an odds ratio of 2.03 indicates that when the student is a non-Buddhist; the tendency for having ED increases. Current OGPA is having a negative relationship with ED, as proved by its negative coefficient of -0.55. Expected degree class contributes positively for having ED among students, suggested by positive coefficient (0.08) and odds ratio (1.09), as seen in Table 11. Further, the increment of family income class increases the chance of getting an ED, due to coefficient of 0.10. As shown in bold fonts in Table 11, the academic stressors 2, 3, 5, 8, 9, 10, 11, and 14 and environmental stressors 1, 2, 5, 6, 7, and 8 have contributed positively for the prevalence of ED in students, and thus can be considered as risk factors for ED.

#### Factors contributing for OCD

Binary logistic regression coefficients, odds ratio, *p*-value, and 95 % confidence interval for each of the predictors of OCD are given in Table 12.

**Table 12:** Binary logistic regression coefficient, odds ratio, *p*-value, and 95% confidence interval table for predicting OCD.

Predictor	Coefficient	Odds ratio	*p*-value	95% CI	Predictor	Coefficient	Odds ratio	*p*-value	95% CI
Gender	0.11	1.12	0.087	[0.28,4.50]	Age	− 0.02	0.98	0.095	[0.45,2.11]
Civil status	− 18.69	0.00	1.000	[0,inf]	Ethnicity	− 0.46	0.63	0.057	[0.13,3.07]
Religion	0.53	1.70	0.035	[0.56,5.14]	Academic year	0.56	1.76	0.029	[0.62,5.03]
Current OGPA	− 1.75	0.17	0.001	[0.04,0.71]	Degree class	− 0.38	0.68	0.030	[0.33,1.41]
Income	0.12	1.12	0.036	[0.87,1.45]	AS1	− 0.49	0.62	0.022	[0.28,1.35]
AS2	− 0.44	0.65	0.035	[0.26,1.62]	**AS3**	0.28	1.32	0.040	[0.69,2.51]
**AS4**	0.39	1.47	0.041	[0.59,3.65]	AS5	− 0.25	0.78	0.058	[0.32,1.90]
**AS6**	0.46	1.58	0.017	[0.83,3.01]	AS7	− 0.24	0.79	0.061	[0.31,1.98]
AS8	− 0.24	0.79	0.055	[0.36,1.71]	**AS9**	0.06	1.06	0.090	[0.45,2.06]
**AS10**	0.28	1.32	0.041	[0.68,2.56]	AS11	− 0.05	0.95	0.090	[0.41,2.20]
AS12	− 1.09	0.33	0.007	[0.10,1.09]	**AS13**	0.34	1.41	0.039	[0.64,3.09]
**AS14**	0.11	1.11	0.074	[0.60,2.06]	ES1	− 0.17	0.85	0.058	[0.46,1.54]
**ES2**	0.38	1.47	0.026	[0.76,2.84]	ES3	− 0.04	0.96	0.090	[0.55,1.69]
ES4	− 0.14	0.87	0.051	[0.58,1.31]	ES5	− 0.44	0.64	0.012	[0.37,1.12]
ES6	− 0.27	0.76	0.032	[0.44,1.31]	**ES7**	0.71	2.03	0.003	[1.07,3.87]
**ES8**	0.18	1.20	0.058	[0.63,2.27]	**ES9**	0.15	1.16	0.074	[0.47,2.88]
ES10	− 0.14	0.87	0.054	[0.56,1.35]	ES11	− 0.84	0.43	0.001	[0.23,0.81]

As seen in Table 12, BLR yields a coefficient of 0.11 and an odds ratio of 1.12 for the gender, concluding that there is 12% higher chance for finding OCD in males than females. Probability of finding OCD seems to reduce with the increment of age, as coefficient being negative (-0.02) and having odds ratio of 0.98. However, with academic year, the probability of finding OCD increases by 76%. A correlation between the prevalence of OCD and the civil status cannot be obtained from BLR, due to the high statistical non-significance suggested by *p*-value of 1.0 for the variable civil status. As odds ratio for ethnicity is 0.63, there is a low possibility of occurring OCD when the race is not Sinhalese. A positive coefficient (0.53) in religion with an odds ratio of 1.70 indicates that when the student is a non-Buddhist; the tendency for having OCD increases. Current OGPA and degree class contribute negatively for having OCD among students, suggested by negative coefficients and less than one odds ratios, as seen in Table 12. Presence of OCD increases with the increment of family income as the coefficient is positive (0.12). As shown in bold fonts in Table 12, the academic stressors 3, 4, 6, 9, 10, 13, and 14 and environmental stressors 2,7,8, and 9 have contributed positively for the prevalence of OCD in students, and thus can be considered as risk factors for OCD.

#### Factors contributing for schizophrenia

Binary logistic regression coefficients, odds ratio, *p*-value, and 95 % confidence interval for each of the predictors of schizophrenia are given in Table 13.

**Table 13:** Binary logistic regression coefficient, odds ratio, *p*-value, and 95% confidence interval table for predicting schizophrenia.

Predictor	Coefficient	Odds ratio	*p*-value	95% CI	Predictor	Coefficient	Odds ratio	*p*-value	95% CI
Gender	− 1.42	0.24	0.008	[0.05,1.18]	Age	− 0.16	0.85	0.069	[0.38,1.89]
Civil status	− 19.41	0.00	1.000	[0,inf]	Ethnicity	1.23	3.41	0.013	[0.69,16.72]
Religion	− 0.63	0.53	0.031	[0.16,1.78]	Academic year	− 0.24	0.79	0.065	[0.28,2.21]
Current OGPA	− 0.31	0.73	0.057	[0.25,2.15]	Degree class	0.04	1.05	0.091	[0.50,2.20]
Income	0.32	1.38	0.002	[1.05,1.80]	**AS1**	0.25	1.29	0.053	[0.58,2.84]
**AS2**	0.10	1.11	0.082	[0.47,2.64]	AS3	− 0.09	0.92	0.081	[0.45,1.85]
AS4	− 0.18	0.83	0.072	[0.31,2.24]	AS5	− 0.45	0.64	0.037	[0.24,1.72]
**AS6**	0.08	1.08	0.082	[0.56,2.09]	AS7	− 0.15	0.86	0.075	[0.33,2.33]
AS8	− 0.17	0.84	0.069	[0.36,1.99]	AS9	− 0.06	0.94	0.089	[0.41,2.18]
AS10	− 0.17	0.84	0.062	[0.42,1.68]	**AS11**	0.83	2.29	0.008	[0.90,5.80]
**AS12**	0.03	1.03	0.096	[0.33,3.19]	AS13	− 0.08	0.92	0.084	[0.40,2.10]
**AS14**	0.62	1.86	0.007	[0.94,3.66]	**ES1**	0.60	1.82	0.011	[0.88,3.76]
**ES2**	0.46	1.58	0.020	[0.79,3.19]	**ES3**	0.20	1.22	0.052	[0.67,2.23]
**ES4**	0.23	1.26	0.031	[0.81,1.99]	**ES5**	0.35	1.41	0.022	[0.81,2.46]
ES6	− 0.59	0.56	0.009	[0.28,1.10]	ES7	− 0.18	0.84	0.052	[0.49,1.42]
**ES8**	0.27	1.31	0.046	[0.64,2.70]	ES9	− 0.88	0.42	0.012	[0.14,1.25]
ES10	− 0.13	0.87	0.061	[0.52,1.46]	ES11	− 0.04	0.96	0.090	[0.52,1.79]

As seen in Table 13, BLR yields a coefficient of -1.42 and an odds ratio of 0.24 for the gender, concluding that there is 76% higher chance for finding schizophrenia in females than males. Probability of finding schizophrenia seems to reduce with the increment of age, as coefficient being negative (-0.16) and having odds ratio of 0.85. Also with academic year, the probability of finding schizophrenia decreases by 21%. A correlation between the prevalence of schizophrenia and the civil status cannot be obtained from BLR, due to the high statistical non-significance suggested by *p*-value of 1.0 for the variable civil status. As odds ratio for ethnicity is 3.41, there is a high possibility of occurring schizophrenia when the race is not Sinhalese. A negative coefficient (-0.63) in religion with an odds ratio of 0.53 indicates that when the student is a non-Buddhist; the tendency for having schizophrenia decreases. Current OGPA also contributes negatively for having schizophrenia among students, suggested by negative coefficient (-0.31) and odds ratio of 0.73, as seen in Table 13. Schizophrenia tends to increase by 5% with increment of the degree class, as suggested by odds ratio of 1.05. Further, increment of family income also tends to increase the chance of having schizophrenia in students. As shown in bold fonts in Table 13, the academic stressors 1, 2, 6, 11, 12, and 14 and environmental stressors 1, 2, 3, 4, 5, and 8 have contributed positively for the prevalence of schizophrenia in students, and thus can be considered as risk factors for schizophrenia.

#### Factors contributing for paranoia

Binary logistic regression coefficients, odds ratio, *p*-value, and 95 % confidence interval for each of the predictors of paranoia are given in Table 14.

**Table 14:** Binary logistic regression coefficient, odds ratio, *p*-value, and 95% confidence interval table for predicting paranoia.

Predictor	Coefficient	Odds ratio	*p*-value	95% CI	Predictor	Coefficient	Odds ratio	*p*-value	95% CI
Gender	− 0.25	0.78	0.075	[0.18,3.44]	Age	0.05	1.06	0.090	[0.45,2.47]
Civil status	− 17.48	0.00	1.000	[0,inf]	Ethnicity	− 2.43	0.09	0.008	[0.01,1.40]
Religion	3.03	20.61	0.000	[2.53,168.21]	Academic year	− 0.02	0.98	0.097	[0.30,3.15]
Current OGPA	0.41	1.51	0.049	[0.47,4.90]	Degree class	0.02	1.02	0.096	[0.39,2.67]
Income	0.26	1.30	0.007	[0.98,1.73]	AS1	− 0.4	0.67	0.039	[0.27,1.67]
AS2	− 0.65	0.52	0.020	[0.19,1.41]	**AS3**	0.11	1.11	0.079	[0.52,2.37]
AS4	− 0.09	0.92	0.086	[0.36,2.36]	**AS5**	0.21	1.23	0.064	[0.52,2.92]
**AS6**	0.77	2.16	0.005	[0.98,4.73]	**AS7**	0.12	1.13	0.082	[0.39,3.62]
AS8	− 0.01	0.99	0.098	[0.41,2.41]	AS9	− 0.24	0.78	0.048	[0.33,1.85]
**AS10**	0.78	2.18	0.008	[0.91,5.21]	**AS11**	0.33	1.39	0.049	[0.49,3.92]
AS12	− 1.29	0.28	0.006	[0.07,1.03]	**AS13**	0.66	1.93	0.022	[0.67,5.55]
AS14	− 0.12	0.89	0.075	[0.44,1.81]	**ES1**	0.13	1.13	0.074	[0.53,2.41]
**ES2**	0.15	1.16	0.073	[0.49,2.74]	ES3	− 0.2	0.82	0.047	[0.44,1.53]
**ES4**	0.19	1.21	0.041	[0.77,1.91]	ES5	− 0.55	0.58	0.006	[0.32,1.03]
ES6	− 0.3	0.74	0.037	[0.39,1.42]	**ES7**	0.63	1.88	0.007	[0.95,3.73]
ES8	− 0.59	0.55	0.012	[0.26,1.18]	ES9	− 0.78	0.46	0.017	[0.15,1.41]
ES10	− 0.42	0.66	0.016	[0.36,1.18]	**ES11**	0.12	1.13	0.071	[0.59,2.16]

As seen in Table 14, BLR yields a coefficient of -0.25 and an odds ratio of 0.78 for the gender, concluding that there is 22% lesser chance for finding paranoia in males than females. Probability of finding paranoia seems to increase with the increment of age, as coefficient being positive (0.05) and having odds ratio of 1.06. However, with academic year, the probability of finding paranoia decreases by 2%. A correlation between the prevalence of paranoia and the civil status cannot be obtained from BLR, due to the high statistical non-significance suggested by *p*-value of 1.0 for the variable civil status. As odds ratio for ethnicity is 0.09, there is a low possibility of occurring paranoia when the race is not Sinhalese. A positive coefficient (3.03) in religion with an odds ratio of 20.61 indicates that when the student is a non-Buddhist; the tendency for having paranoia increases dramatically. Current OGPA and degree class also contribute positively for having paranoia among students, suggested by positive coefficients and large odds ratios, as seen in Table 14. Increment of the family income also contributes positively for the presence of paranoia in students, as suggested by the positive coefficient (0.26). As shown in bold fonts in Table 14, the academic stressors 3, 5, 6, 7, 10, 11, and 13 and environmental stressors 1, 2, 4, 7, and 11 have contributed positively for the prevalence of paranoia in students, and thus can be considered as risk factors for paranoia.

#### Factors contributing for PTSD

Binary logistic regression coefficients, odds ratio, *p*-value, and 95 % confidence interval for each of the predictors of PTSD are given in Table 15.

**Table 15:** Binary logistic regression coefficient, odds ratio, *p*-value, and 95% confidence interval table for predicting PTSD.

Predictor	Coefficient	Odds ratio	*p*-value	95% CI	Predictor	Coefficient	Odds ratio	*p*-value	95% CI
Gender	15.0	3.25E06	0.064	[0.41,4.62E11]	Age	− 3.50	0.03	0.027	[0.00,0.85]
Civil status	34.36	8.36E14	1.000	[0.00,inf]	Ethnicity	3.19	24.26	0.068	[0.63,148.2]
Religion	− 3.47	0.03	0.017	[0.00,0.73]	Academic year	1.47	4.34	0.097	[0.72,85.32]
Current OGPA	3.17	23.83	0.032	[0.54, 278.8]	Degree class	− 3.37	0.03	0.069	[0.00,0.93]
Income	0.78	2.19	0.074	[0.71,56.2]	**AS1**	0.44	1.55	0.082	[0.61,18.2]
AS2	− 1.56	0.21	0.052	[0.01,1.21]	AS3	− 4.28	0.01	0.080	[0.00,0.85]
**AS4**	4.47	87.7	0.051	[0.67,243.2]	AS5	− 10.01	0.00	0.013	[0.00,0.35]
**AS6**	9.89	1.97E04	0.011	[0.43,7.21E10]	**AS7**	3.20	24.51	0.058	[0.54,106.3]
AS8	− 7.81	0.00	0.001	[0.00,0.28]	**AS9**	11.81	1.35E05	0.064	[0.26,4.15E12]
**AS10**	8.9	7.36E03	0.010	[0.48,2.39E08]	AS11	− 4.39	0.01	0.011	[0.00,0.19]
AS12	− 3.51	0.03	0.075	[0.00,0.42]	**AS13**	3.46	31.68	0.020	[0.56,142.3]
**AS14**	1.62	5.03	0.062	[0.37,58.3]	ES1	− 2.72	0.07	0.045	[0.01,0.85]
**ES2**	5.77	319.23	0.007	[0.84,821.3]	ES3	− 3.14	0.04	0.041	[0.00,0.93]
ES4	− 0.8	0.45	0.003	[0.02,1.12]	ES5	− 0.79	0.45	0.002	[0.02,0.97]
**ES6**	0.08	1.08	0.056	[0.47,10.52]	ES7	− 2.59	0.08	0.003	[0.01,0.87]
**ES8**	0.97	2.65	0.015	[0.37,9.21]	**ES9**	4.76	116.48	0.048	[0.79,303.6]
**ES10**	1.76	5.83	0.006	[0.68,15.2]	ES11	− 1.52	0.22	0.015	[0.02,1.07]

As seen in Table 15, BLR yields a coefficient of 15.0 and an odds ratio of 3.25E06 for the gender, concluding that there is a very higher chance for finding PTSD in males than females. Probability of finding PTSD seems to reduce with the increment of age, as coefficient being negative (-3.50) and having odds ratio of 0.03. However, with academic year, the probability of finding PTSD increases by 334%. A correlation between the prevalence of PTSD and the civil status cannot be obtained from BLR, due to the high statistical non-significance suggested by *p*-value of 1.0 for the variable civil status. As odds ratio for ethnicity is 24.26, there is a very high possibility of occurring PTSD when the race is not Sinhalese. A negative coefficient (-3.47) in religion with an odds ratio of 0.03 indicates that when the student is a non-Buddhist; the tendency for having PTSD decreases. Current OGPA also contributes positively for having PTSD among students, suggested by positive coefficient and large odds ratio, as seen in Table 15. But, the increment of the degree class causes PTSD presence to decrease, suggested by negative coefficient of -3.37. Increment of family income class tends to increase the probability of finding PTSD. As shown in bold fonts in Table 15, the academic stressors 1, 4, 6, 7, 9, 10, and 13 and environmental stressors 2, 6, 8, 9, and 10 have contributed positively for the prevalence of PTSD in students, and thus can be considered as risk factors for PTSD.

#### Factors contributing for psychosis

Binary logistic regression coefficients, odds ratio, *p*-value, and 95 % confidence interval for each of the predictors of psychosis are given in Table 16.

**Table 16:** Binary logistic regression coefficient, odds ratio, *p*-value, and 95% confidence interval table for predicting psychosis.

Predictor	Coefficient	Odds ratio	*p*-value	95% CI	Predictor	Coefficient	Odds ratio	*p*-value	95% CI
Gender	0.51	1.67	0.049	[0.34,8.27]	Age	− 0.06	0.94	0.088	[0.44,2.04]
Civil status	− 19.12	0.00	1.000	[0,inf]	Ethnicity	− 0.64	0.53	0.049	[0.08,3.60]
Religion	0.83	2.30	0.012	[0.80,6.57]	Academic year	0.20	1.23	0.071	[0.42,3.59]
Current OGPA	− 0.26	0.77	0.062	[0.28,2.14]	Degree class	0.15	1.17	0.069	[0.54,2.49]
Income	− 0.02	0.98	0.086	[0.76,1.26]	AS1	− 0.05	0.95	0.091	[0.43,2.09]
AS2	− 0.63	0.53	0.017	[0.22,1.30]	**AS3**	0.44	1.56	0.022	[0.77,3.14]
AS4	− 0.18	0.83	0.069	[0.34,2.04]	AS5	− 0.24	0.78	0.057	[0.34,1.79]
**AS6**	0.88	2.41	0.002	[1.14,5.11]	**AS7**	0.09	1.09	0.085	[0.42,2.85]
AS8	− 0.93	0.39	0.002	[0.18,0.89]	AS9	− 0.2	0.82	0.062	[0.37,1.80]
**AS10**	0.09	1.10	0.078	[0.56,2.15]	**AS11**	0.40	1.49	0.040	[0.58,3.82]
AS12	− 0.05	0.96	0.093	[0.34,2.65]	**AS13**	0.41	1.51	0.029	[0.71,3.23]
**AS14**	0.01	1.01	0.097	[0.56,1.81]	**ES1**	0.32	1.37	0.036	[0.69,2.72]
ES2	− 0.05	0.95	0.089	[0.47,1.93]	**ES3**	0.22	1.25	0.049	[0.67,2.32]
**ES4**	0.41	1.50	0.008	[0.96,2.36]	ES5	− 0.57	0.56	0.004	[0.32,0.98]
ES6	− 0.14	0.87	0.062	[0.50,1.51]	ES7	− 0.15	0.86	0.048	[0.50,1.48]
**ES8**	0.11	1.11	0.075	[0.58,2.13]	ES9	− 0.47	0.63	0.030	[0.26,1.52]
**ES10**	0.15	1.16	0.046	[0.74,1.82]	**ES11**	0.29	1.34	0.033	[0.74,2.41]

As seen in Table 16, BLR yields a coefficient of 0.51 and an odds ratio of 1.67 for the gender, concluding that there is 67% higher chance for finding psychosis in males than females. Probability of finding psychosis seems to reduce with the increment of age, as coefficient being negative (-0.06) and having odds ratio of 0.94. However, with academic year, the probability of finding psychosis increases by 23%. A correlation between the prevalence of psychosis and the civil status cannot be obtained from BLR, due to the high statistical non-significance suggested by *p*-value of 1.0 for the variable civil status. As odds ratio for ethnicity is 0.53, there is a low possibility of occurring psychosis, when the race is not Sinhalese. A positive coefficient (0.83) in religion with an odds ratio of 2.30 indicates that when the student is a non-Buddhist; the tendency for having psychosis increases. Current OGPA increment tends to decrease the probability of the presence of psychosis in students, as suggested by the negative coefficient of -0.26. Degree class contributes positively for having psychosis among students, suggested by positive coefficient and large odds ratio, as seen in Table 16. Increment of family income class tends to decrease the prevalence of psychosis among students. As shown in bold fonts in Table 16, the academic stressors 3, 6, 7, 10, 11, 13, and 14 and environmental stressors 1, 3, 4, 8, 10, and 11 have contributed positively for the prevalence of psychosis in students, and thus can be considered as risk factors for psychosis.

## Discussion

In this section, we will discuss the cross analysis of factors causing psychiatric illnesses among students, in order to identify the risk factors for causing psychiatric illnesses. For this purpose, we consider the sign of the BLR coefficient of a given predictor for BLR results of all 13 psychiatric illnesses. In other words, we examine the sign of BLR coefficient of a given predictor among all BLR tables from Tables 4, 5, 6, 7, 8, 9, 10, 11, 12, 13, 14, 15, 16. After examining, we count the number of diseases which the BLR coefficient was positive, and use Eq. 16 to calculate the risk percentage (R). For 9 psychiatric illnesses, the prevalence was higher for the male gender. Only for PD, SAD, schizophrenia, and paranoia; the probability of finding the illness was higher among the feminine gender. Thus, the risk percentage (R) for male gender is 69%. In almost all diseases, except for the cases of ED and depression, the possibility of prevalence of the psychiatric illness has reduced with the increment of age. Thus, the risk percentage for being in a lesser age group is 77%. Age is not directly related with the academic year, because for a given academic year, there can be students with multiple ages such as first shy students, second shy students, third shy students, repeat or batch missed students, etc. Increment of academic year showed positive signs for chances of prevalence of psychiatric illness, except for the cases of depression, schizophrenia, and paranoia. Thus, the risk percentage for being in a higher academic year is 77%. When the students go high in academic years, their workload and expected cognitive skills increase; so, it is justifiable why for most of the illnesses, advancement of the academic year contributes for finding psychiatric illnesses more. A relationship between the prevalence of a psychiatric illness and the civil status could not be obtained, due to the high statistical non-significance of the variable civil status in BLR. Most of the psychiatric illnesses were found in Sinhalese, except for the cases of agoraphobia, PD, DD, schizophrenia, and PTSD. Thus, the risk percentage for being a Sinhalese is 72%. This can also be because of the fact that majority of the population were Sinhalese. However, even though most of the student population were Buddhists, only 4 psychiatric illnesses (agoraphobia, DD, schizophrenia, PTSD) were more probable among Buddhists. Thus, the risk percentage for being a non-Buddhist is 69%. Both current OGPA and expected degree class affected for the presence of psychiatric illnesses in a mixed manner. For some diseases, they had a positive relationship, and vice versa. But it was very evident that GAD and paranoia were probable in those students with a high OGPA, who are expecting a high-class degree. Further, for those students expecting a high-class degree; DD, ED, schizophrenia, and psychosis were more probable. Surprisingly, the increment of family income or in other terms, students from rich family backgrounds have a higher probability of prevalence of psychiatric illnesses than students from poor families, except for the cases of GAD, SAD, and psychosis. Thus, the risk percentage (R) for being in a high-income family class is 77%. This can be argued due to the fact that richer students who have been accustomed for a comfortable and caring life from their childhood being less tolerable for the stressors, once become students under academic and environmental stressors, tend to suffer from psychiatric illnesses.

The factor “being satisfied in the degree program” (AS1) negatively affects the presence of psychiatric illnesses, except for the cases of PD, schizophrenia and PTSD. So, it is very clear that students those who are less satisfied about the degree program tend to suffer from many psychiatric illnesses such as depression, BAD, agoraphobia, etc. Next, conventional written examinations (AS2) do not seem to contribute much positively for the prevalence of mental illnesses, except for the cases of SAD, ED, and schizophrenia. On the other hand, online written end semester/year-end exams (AS3) contribute positively for the prevalence of many psychiatric illnesses, except for the cases of GAD, BAD, schizophrenia, and PTSD. Thus, the risk percentage for facing online written examination is 69%. This proves that online mode of evaluation tends to increase the psychiatric illnesses among students. Oral examinations (viva - AS4) also tend to increase mental illnesses, except for the cases of ED, schizophrenia, paranoia, and psychosis. Thus, the risk percentage for oral examination is also 69%. Oral presentation (AS5) does not seem to contribute positively for the prevalence of mental illnesses, except for the cases of BAD, DD, ED, and paranoia. Individual self-learning (AS6) tends to increase the prevalence of almost all the mental illnesses, except ED. Thus, the risk percentage for individual self-learning is very high as 92%. It is difficult to predict whether participation for a lecture (AS7) tends to increase the mental illnesses in overall, because it has mixed impact on the prevalence of illnesses. Participation for an online lecture (AS8) does not increase the tendency for occurrence of psychiatric illnesses in most cases, except for the cases of agoraphobia, BAD, ED, and DD. Active learning (AS9) tends to increase most of the prevalence of mental illnesses, except for the cases of schizophrenia, paranoia, and psychosis. Thus, the risk percentage for active learning is 77%. Research and project development work (AS10) also tends to increase the prevalence of most of the mental illnesses, except for the cases of DD and schizophrenia. Thus, the risk percentage for research and development work is 85%. It is difficult to predict the overall effect of the online quiz (AS11), as it contributes in both ways (presence or absence) of psychiatric illnesses. Physical in-class tests (AS12) do not seem to contribute for the presence of most of the psychiatric illnesses, except for the cases of PD, SAD, and schizophrenia. Practical demonstrations (AS13) also tend to increase the presence of most of the mental illnesses, except for the cases of SAD, BAD, ED, and schizophrenia. Thus, the risk percentage for practical demonstrations is 69%. Industrial/professional/worksite training (AS14) also contributes positively for the prevalence of most of the psychiatric illnesses, except for the cases of GAD, PD, depression, and paranoia. Thus, the risk percentage for Industrial/professional/worksite training is also 69%.

Prevalence of financial difficulties (ES1) has also contributed positively for the prevalence of psychiatric illnesses, except in cases of agoraphobia, depression, DD, OCD, and PTSD. Thus, the risk percentage for financial difficulties is 62%. COVID-19 (ES2) has very clearly contributed for the prevalence of psychiatric illnesses, as only 2 cases (PD and psychosis) were otherwise. Thus, the risk percentage for the prevalence of COVID-19 is 85%. Presence of a physical illness (ES3) also had contributed for the presence of most of the psychiatric illnesses, except for the cases of agoraphobia, ED, OCD, paranoia, and PTSD. Thus, the risk percentage for presence of a physical illness is 62%. Relationship problems (ES4) also clearly contribute for the increment of most of the mental illnesses, except for the cases of BAD, ED, OCD, and PTSD. Thus, the risk percentage for the presence of relationship problems is 62%. Bad experience due to ragging (ES5) was not a risk factor for most of the illnesses, but it was a risk factor for the agoraphobia, PD, BAD, ED, and schizophrenia. This verifies the previously obtained result that for most of the students, PTSD is not caused due to ragging. Death or sickness of a close associate (ES6) was not a risk factor for most of the illnesses, except for GAD, SAD, DD, and PTSD. Accommodation problems (ES7) had a mixed effect for the presence or absence of psychiatric illnesses. Problems in the teaching learning process (ES8) clearly had a positive effect for the presence of most of the psychiatric illnesses, except for the cases of GAD, SAD, depression, and paranoia. Thus, the risk percentage for problems in teaching learning process is 69%. Troubles in online learning (ES9) was not a positively contributing factor for most of the psychiatric illnesses, except for PD, SAD, OCD, and PTSD. Lack of support for psychiatric help (ES10) had a mixed impact for the presence or absence of psychiatric illnesses. Having less or no time to spend for leisure/sports/music etc. (ES11) also had a mixed impact for the presence or absence of psychiatric illnesses, so overall effect cannot be decided.

Now, let us compare our results with existing literature. Our results agree with the fact that online proctoring of examinations has caused anxiety in students as shown in study^7^ ; as we proved that written online examination (AS3) causes stress in 63% of the students having a R value of 69 %. According to research conducted in^4^, it has been found that COVID-19 (ES3) has caused high level of anxiety among students. We also proved that COVID-19 causes stress in 34% of the students, and identified it as a risk factor with a R value of 85%. Agreeing with the fact that health anxiety causes distress in students as mentioned in the work of^6^; our results proved that presence of a physical illness (ES3) causes stress in 17% students, and identified it as a risk factor with a R value of 62%. Even though it has been found that active learning can increase or decrease anxiety among students, according to the way it is implemented as given in the research work of^5^; we found out that active learning (AS9) causes stress in 14% of students, and identified it as a risk factor with a R value of 79%. According to research conducted in^8^, it has been found that female students tend to have higher anxiety than male students, whereas our result on SAD agrees with this finding; but results on GAD, agoraphobia disagree with previous finding. According to the study done in^13^, there is no evidence to prove whether agoraphobia existed in students or not. However, we have successfully proved that 27% [18.30, 35.70] students were screened positive for agoraphobia, and identified individual risk factors also. Therefore, we are one of the first to investigate on prevalence of agoraphobia among the students. There is ample evidence for existence of the panic disorder also, as highlighted in research work of^14^. We have successfully proved that PD exists among students with a prevalence of 14% [7.19,20.80], and identified individual risk factors for the disease also. Our results strongly agree with the findings of the research conducted in^15^  which they have found a high prevalence for SAD; as we also found that SAD with the highest prevalence percentage of 50% [40.2, 59.8] in the sample of students in university of Ruhuna. We found SAD more in female students (BLR coefficient of − 1.43 and odds ratio of 0.24) after a BLR analysis, and this fact agrees with the findings of the work in^16^ which they have found SAD more dominant in feminine gender, in a sample of Egyptian university students. Research conducted in^17^  has found an association between SAD and depression; and we also found a medium correlation between SAD and depression with a Pearson correlation coefficient of 0.32. We found nearly half (46% with a 95% CI of [36.23,55.77]) of the students screened positive for depression, whereas according to study in^19^; nearly one third of the students have depression in average. This shows that the prevalence of depression in our sample is high. Even though it has been found that 10% of medical students has depression to the level of suicidal ideation according to studies done in^21,28^; we only found 6% with severe depression in our sample, possibly because our sample contains students from multiple disciplines. We found that after a BLR analysis that there is 163% higher chance in finding depression in male gender than female gender, which agrees with the findings of research conducted in^8^. Research done in^29^  shows that only few Sri Lankan undergraduates who have psychiatric illnesses seek professional support; whereas our findings also show that only 8% have sought medical advise from psychiatrists. Recent studies^30,31^  suggest that BAD prevalence is lesser than that of anxiety, and our results also depict a low prevalence percentage of only 8% [2.68, 13.32] for the BAD; which is lower than all anxiety related disorders such as GAD (36%), agoraphobia (27%), and SAD (50%). Even though some points out that there is no significant relationship between GPA and BAD according to study in^33^ ; we have found out a strong positive correlation (BLR coefficient of 8.54 and odds ratio of 5110) between the academic performance (OGPA) and prevalence of BAD. We found DD in our sample with a prevalence percentage of 11% [4.87, 17.13], similar to the studies conducted in works^34,35^. In a group of Malaysian students, 14% had been screened positive for ED according to research done in^42^; whereas in our sample, we found 36% [26.59, 45.41] screened positive for ED. Some studies such as^43,44^  have found ED more dominant in the feminine gender; however according to BLR analysis for our sample (BLR coefficient of 0.70 and odds ratio of 2.02), we identified it more among the male gender. In our sample, 34% [24.72,43.28] students screened positive for OCD, similar to the group of college students in Kerala^48^. Our result for OCD showed that there is a strong relationship between fear of COVID-19 (ES2) and presence of OCD; as proved by BLR coefficient of 0.38 and odds ratio of 1.47, which verifies the study^50^  which highlights that fear of COVID-19 has caused students to develop OCD symptoms. Research work in^54^  shows that there is only a probability of 0.03 for the co-occurrence of schizophrenia and OCD. As suggested by the Pearson correlation coefficient of 0.05 between schizophrenia and OCD; our results also show that there is only a low probability of co-occurrence of schizophrenia and OCD. Studies such as^56^  show that transgender students tend to suffer more from schizophrenia than cisgender students. Unfortunately, our sample did not contain any transgender students, so that we could not come to any conclusion regarding the relationship between transgender students and prevalence of schizophrenia. Agreeing with works of^57,58^  which prove that there is high prevalence of paranoia among students; our sample also had a high prevalence percentage of 42% [32.33, 51.67]. We found students screened positive for PTSD with a low percentage of 3% [0.00,6.34], similar to the studies done in^61,62^. According to research conducted using a group of British undergraduate students; financial difficulties has been a major contributor to psychosis risk^65^. Our results also proved that financial difficulties (ES1) positively contribute for the prevalence of psychosis, as evident from the BLR coefficient of 0.32 and odds ratio of 1.37. Study in^67^  shows depression as a risk factor for psychosis, and we have also found a moderate association between psychosis and depression, as proved by the Pearson correlation coefficient of 0.36. According to the study in^69^; students with psychosis tend to have poor cognitive function. This fact is proved in our study which shows that there is a negative correlation between OGPA and prevalence of psychosis; as BLR analysis yielded a coefficient of -0.26 and odds ratio of 0.77 for the correlation between OGPA and psychosis.

We have studied on the factors, remedies and prevalence of psychiatric illnesses among a random sample of students in university of Ruhuna, which is a Sri Lankan university. The specialty in our study is that we do not limit our study to a set of students of a particular discipline (subject area for example medicine). Our sample contains students from multiple disciplines. The outcomes may vary based on demographic factors, environmental factors, and academic factors in other universities of the world. But in our findings, especially in prevalence and for odds ratio value in identifying factors contributing for a particular psychiatric illness using BLR, we have specified 95% confidence intervals, so that we can expect the outcomes to vary in the specified intervals. Our results highly agree with similar work in existing literature as discussed above, suggesting that even though our case study involves students only from the university of Ruhuna, we can expect similar outcomes for students in other universities of the world. This research is done for screening patients for a particular psychiatric illness and for preliminary investigation only, and does not involve a clinical diagnosis by a trained psychiatrist. However, preliminary investigation results will be very useful to recognize the requirement to provide psychiatric assistance to students in need, and to change the educational policies to reduce the associated risks.

## Recommendations for policymakers

The universities must consider the environmental and academic risk factors associated with psychiatric illnesses, and design curriculum to reduce the risk factors.

- As 63% of students have felt the highest stress for the online written examination (AS3) and it has a risk percentage (R) of 69%, the policymakers should avoid conducting online written examinations as much as possible.
- As only 15% get stressed in participating for online classes (AS8) and has a low R value, authorities may continue the online mode of delivering lectures.
- As higher percentage of students feel stressed when engaging in active learning (AS9) than passive learning of participating in a physical lecture (AS7), and active learning has R value of 79%, authorities may conduct most of their teaching in passive mode.
- Other modes of examination such as viva and practical demonstrations must be conducted in such a manner that students feel relaxed and friendly, as both of these examinations are risk factors having high risk percentages for prevalence of psychiatric illnesses.
- Authorities must expand resources such as laboratory facilities, online support, financial support, supervision, and academic instruction assistance for research and development work and individual self learning work, as they have been identified as risk factors.
- As 25% of the students get stressed due to financial difficulties, and it has been identified as a risk factor with R value of 62% the authorities must take actions to provide financial assistance to the students.
- Further as 61% of the students mention that they don’t receive psychiatric help from the university, and the presence of a physical illness and relationship problems have been identified as risk factors; authorities must expand resources for providing resources for sports, music, cultural event organizing, and provide counseling services.

## Conclusion

All 13 psychiatric illnesses were found in the sample of students with a per disease mean prevalence percentage of 28, having a standard deviation of 14.36, despite the prevalence of well-being factors among students, and only 8% are clinically diagnosed. There was no strong correlation between presence of each psychiatric illness; but medium correlation was observed between some pairs of illnesses. Students are mainly using the remedies: relaxation techniques (Music, sports, leisure, etc.), support from family and friends, online social networks, mindfulness meditation, and online gaming to reduce the psychological distress. Individual risk factors for each of the psychiatric illnesses were identified after a BLR analysis. Finally, a conclusion on overall risk factors for the presence of a psychiatric illness in general can be derived as follows. Being a male (R = 69%), lesser age group (R = 77%), Sinhalese (R = 72%), Non-Buddhist (R = 69%), and having a high family income class (R = 77%) were identified as demographic risk factors for the presence of a psychiatric illness. Being in a high academic year (R = 77%), having to face online written end examination (R = 69%), having to face oral examination (R = 69%), individual self-learning (R = 92%), active learning involving group work (R = 77%), research and development work (R = 85%), practical demonstrations (R = 69%), and industrial/professional/worksite training (R = 69%) are the academic risk factors contributing positively for the prevalence of psychiatric illnesses. Prevalence of financial difficulties (R = 62%), prevalence of COVID-19 (R=85 %), presence of a physical illness (R = 62%), presence of relationship problems (R = 62%), and problems in the teaching-learning process (R = 69%) were identified as the environmental risk factors. The level of prevalence of psychiatric illnesses was high, as 89% of the students were suffering from at least one psychiatric illness, and 68% were screened to be psychologically distressed.

### Limitations

The case study investigates on the factors, remedies, and prevalence of psychiatric illnesses for a sample of students in a Sri Lankan university. The sample size is only 100, so that there is uncertainty involved in the results. For instance, we had captured and presented the uncertainties of prevalence percentage of psychiatric illnesses as 95% confidence intervals which we can expect the prevalence percentages to vary as evident from Table 2 and Fig. 6. Furthermore, for the binary logistic regression analysis also, when identifying individual risk factors, we specify a 95% confidence interval for the odds ratio to capture the uncertainty of the relationship between the prevalence and individual factor considered, as evident from Tables 4, 5, 6, 7, 8, 9, 10, 11, 12, 13, 14, 15, 16. If a similar study is carried out in another university of the world, the outcomes may vary based on demographic factors, academic factors, and environmental factors in those universities. Standard questionnaires used in the context of this research are used for screening patients for a particular psychiatric illness and for preliminary investigation. Further investigation and a clinical diagnosis by a psychiatrist may be required in order to confirm the presence of a psychiatric illness of a screened patient.

## Data Availability

The datasets analyzed during the current study are not publicly available due ethical reasons but are available from the corresponding author on reasonable request. Datasets are also available for the Journal for reviewing purposes.

## References

[1] Bolliger, D. U. & Halupa, C. Student perceptions of satisfaction and anxiety in an online doctoral program. *Distance Educ.* **33**(1), (2012)

[2] Cornine A (2020). Reducing nursing student anxiety in the clinical setting: An integrative review. Nurs. Educ. Perspect..

[3] Jones PJ, Park SY, Lefevor GT (2018). Contemporary college student anxiety: The role of academic distress, financial stress, and support. J. Coll. Couns..

[4] Ma H, Miller C (2021). Trapped in a double bind: Chinese overseas student anxiety during the COVID-19 pandemic. Health Commun..

[5] Cooper KM, Downing VR, Brownell SE (2018). The influence of active learning practices on student anxiety in large-enrollment college science classrooms. Int. J. STEM Educ..

[6] Kosic A, Lindholm P, Järvholm K, Hedman-Lagerlöf E, Axelsson E (2020). Three decades of increase in health anxiety: Systematic review and meta-analysis of birth cohort changes in university student samples from 1985 to 2017. J. Anxiety Disord..

[7] Woldeab, D. & Brothen, T. 21st Century assessment: Online proctoring, test anxiety, and student performance. *Int. J. E-learn. Distance Educ.* **34**(1), (2019)

[8] Gao W, Ping S, Liu X (2020). Gender differences in depression, anxiety, and stress among college students: A longitudinal study from China. J. Affect. Disord..

[9] Rempel BP, Dirks MB, McGinitie EG (2021). Two-stage testing reduces student-perceived exam anxiety in introductory chemistry. J. Chem. Educ..

[10] Bamber MD, Morpeth E (2019). Effects of mindfulness meditation on college student anxiety: A meta-analysis. Mindfulness.

[11] Anderson D, Brown S (2021). The effect of animal-assisted therapy on nursing student anxiety: A randomized control study. Nurse Educ. Pract..

[12] Griffin M, Campos HC, Khramtsova I, Pearce A (2020). Stress and anxiety reduction in college students through biofeedback. Coll. Stud. J..

[13] Angle, S. P. Perceptions of college students diagnosed with panic disorder with agoraphobia: Academic, psychosocial, and environmental views of their college experience. *Virginia Polytechnic Institute and State University*. Ph.D thesis (1999).

[14] Jurin T, Biglbauer S (2018). Anxiety sensitivity as a predictor of panic disorder symptoms: A prospective 3-year study. Anxiety Stress Coping.

[15] Desalegn GT, Getinet W, Tadie G (2019). The prevalence and correlates of social phobia among undergraduate health science students in Gondar, Gondar Ethiopia. BMC Res. Notes.

[16] Rabie, M. A., Shorab, E., ElGabry, D., Aziz, K. A., Sabry, W. M., Aufa, O., ElGhamry, R., Hassan, G. & Nagy, N. Screening of social phobia symptoms in a sample of Egyptian university students. *Arch. Clin. Psychiatry (São Paulo)* **46**, 27–32 (2019).

[17] Elavarasan K, Dhandapani T, Norman P, Vidya DC, Mani G (2018). The association between internet addiction, social phobia and depression in medical college students. Int. J. Commun. Med. Public Health.

[18] Akram MS, Naik P, Nirgude AS (2015). A study on social phobia and functional disability among university students of Dakshina Kannada district. Hindu.

[19] Ibrahim AK, Kelly SJ, Adams CE, Glazebrook C (2013). A systematic review of studies of depression prevalence in university students. J. Psychiatr. Res..

[20] Puthran, R., Zhang, M. W., Tam, W. W. & Ho, R. C. Prevalence of depression amongst medical students: A meta-analysis. *Med. Educ.* **50**(4), (2016) 10.1111/medu.12962 26995484

[21] Rotenstein LS, Ramos MA, Torre M, Segal JB, Peluso MJ, Guille C, Sen S, Mata DA (2016). Prevalence of depression, depressive symptoms, and suicidal ideation among medical students: A systematic review and meta-analysis. JAMA.

[22] Satinsky EN, Kimura T, Kiang MV, Abebe R, Cunningham S, Lee H, Lin X, Liu CH, Rudan I, Sen S, Tomlinson M (2021). Systematic review and meta-analysis of depression, anxiety, and suicidal ideation among Ph.D. D. students. Sci. Rep..

[23] Beiter R, Nash R, McCrady M, Rhoades D, Linscomb M, Clarahan M, Sammut S (2015). The prevalence and correlates of depression, anxiety, and stress in a sample of college students. J. Affect. Disord..

[24] Wang ZH, Yang HL, Yang YQ, Liu D, Li ZH, Zhang XR, Zhang YJ, Shen D, Chen PL, Song WQ, Wang XM (2020). Prevalence of anxiety and depression symptom, and the demands for psychological knowledge and interventions in college students during COVID-19 epidemic: A large cross-sectional study. J. Affect. Disord..

[25] Rith-Najarian LR, Boustani MM, Chorpita BF (2019). A systematic review of prevention programs targeting depression, anxiety, and stress in university students. J. Affect. Disord..

[26] Lattie EG, Adkins EC, Winquist N, Stiles-Shields C, Wafford QE, Graham AK (2019). Digital mental health interventions for depression, anxiety, and enhancement of psychological well-being among college students: systematic review. J. Med. Internet Res..

[27] Alsubaie MM, Stain HJ, Webster LAD, Wadman R (2019). The role of sources of social support on depression and quality of life for university students. Int. J. Adolesc. Youth.

[28] Amarasuriya SD, Jorm AF, Reavley NJ (2015). Prevalence of depression and its correlates among undergraduates in Sri Lanka. Asian J. Psychiatr..

[29] Amarasuriya, S. D., Reavley, N. J., Rossetto, A. & Jorm, A. F. Helping intentions of undergraduates towards their depressed peers: A cross-sectional study in Sri Lanka. *BMC Psychiatry*. **17**(1), (2017) 10.1186/s12888-017-1192-7 PMC5259849 28114918

[30] Pedersen DE (2020). Bipolar disorder and the college student: A review and implications for universities. J. Am. Coll. Health.

[31] Vargas-Huicochea I, Robles-García R, Berlanga C, Tovilla-Zarate CA, Martínez-López N, Fresán A (2017). Mental health literacy about bipolar disorder and schizophrenia among medical students: A comparative study of illness recognition, treatment, and attitudes according to perception of aggressiveness-dangerousness. Salud Ment..

[32] Shenoy SK, Praharaj SK (2019). Borderline personality disorder and its association with bipolar spectrum and binge eating disorder in college students from South India. Asian J. Psychiatr..

[33] Li, A., Teng, J., Tajchman, Z. J. & Vilares, I. The Relationship between Bipolar and Borderline Personality Disorder traits, Impulsivity, and GPA among a college student population. *PsyArXiv preprints* (2021).

[34] Kate MA, Hopwood T, Jamieson G (2020). The prevalence of dissociative disorders and dissociative experiences in college populations: A meta-analysis of 98 studies. J. Trauma Dissoc..

[35] Fung HW, Ling HH, Ross CA, Tse JL, Liu RKW (2020). Dissociative, Schneiderian and borderline personality symptoms in a non-clinical sample in Hong Kong: A preliminary report. Eur. J. Trauma Dissoc..

[36] De Pasquale C, Dinaro C, Sciacca F (2018). Relationship of Internet gaming disorder with dissociative experience in Italian university students. Ann. Gen. Psychiatry.

[37] Şar V, Türk T, Öztürk E (2019). Fear of happiness among college students: The role of gender, childhood psychological trauma, and dissociation. Indian J. Psychiatry.

[38] Sonneville KR, Lipson SK (2018). Disparities in eating disorder diagnosis and treatment according to weight status, race/ethnicity, socioeconomic background, and sex among college students. Int. J. Eat. Disord..

[39] Lipson SK, Sonneville KR (2017). Eating disorder symptoms among undergraduate and graduate students at 12 US colleges and universities. Eat. Behav..

[40] Zickgraf HF, Hazzard VM, O’Connor SM, Simone M, Williams-Kerver GA, Anderson LM, Lipson SK (2020). Examining vegetarianism, weight motivations, and eating disorder psychopathology among college students. Int. J. Eat. Disord..

[41] Ganson KT, Nagata JM (2021). Associations between vaping and eating disorder diagnosis and risk among college students. Eat. Behav..

[42] Chan YL, Samy AL, Tong WT, Islam MA, Low WY (2020). Eating disorder among Malaysian University students and its associated factors. Asia Pac. J. Public Health.

[43] Blair L, Aloia CR, Valliant MW, Knight KB, Garner JC, Nahar VK (2017). Association between athletic participation and the risk of eating disorder and body dissatisfaction in college students. Int. J. Health Sci..

[44] Stoeber J, Madigan DJ, Damian LE, Esposito RM, Lombardo C (2017). Perfectionism and eating disorder symptoms in female university students: The central role of perfectionistic self-presentation. Eat. Weight Disord. Stud. Anorexia Bulimia Obes..

[45] Solly, J. E., Chamberlain, S. R., Lust, K. & Grant, J. E. Binge-eating disorder in university students: High prevalence and strong link to impulsive and compulsive traits. *CNS Spectrums*, 1–9 (2021) 10.1017/S1092852921000882 PMC7614224 34658319

[46] Cheng PH, Merrick E (2017). Cultural adaptation of dialectical behavior therapy for a Chinese international student with eating disorder and depression. Clin. Case Stud..

[47] Stice E, Rohde P, Shaw H, Gau JM (2017). Clinician-led, peer-led, and internet-delivered dissonance-based eating disorder prevention programs: Acute effectiveness of these delivery modalities. J. Consult. Clin. Psychol..

[48] Jaisoorya TS, Reddy YJ, Nair BS, Rani A, Menon PG, Revamma M, Jeevan CR, Radhakrishnan KS, Jose V, Thennarasu K (2017). Prevalence and correlates of obsessive-compulsive disorder and subthreshold obsessive-compulsive disorder among college students in Kerala, India. Indian J. Psychiatry.

[49] Brytek-Matera A, Fonte ML, Poggiogalle E, Donini LM, Cena H (2017). Orthorexia nervosa: relationship with obsessive-compulsive symptoms, disordered eating patterns and body uneasiness among Italian university students. Eat. Weight Disord. Stud. Anorexia Bulimia Obes..

[50] Ji G, Wei W, Yue KC, Li H, Shi LJ, Ma JD, He CY, Zhou SS, Zhao Z, Lou T, Cheng J (2020). Effects of the COVID-19 pandemic on obsessive-compulsive symptoms among university students: Prospective cohort survey study. J. Med. Internet Res..

[51] Liu W, Li J, Huang Y, Yu B, Qin R, Cao X (2021). The relationship between left-behind experience and obsessive-compulsive symptoms in college students in China: The mediation effect of self-esteem. Psychol. Health Med..

[52] Woerner M, Selles RR, De Nadai AS, Salloum A, Storch EA (2017). Hoarding in college students: Exploring relationships with the obsessive compulsive spectrum and ADHD. J. Obsessive Compulsive Relat. Disord..

[53] Wheaton MG, Gallina ER (2019). Using cognitive-behavioral therapy to treat obsessive-compulsive disorder with co-occurring depression. J. Cogn. Psychother..

[54] Shan HD, Zhang RT, Jiang SY, Wang YM, Liu YF, Cheung EF, Chan RC (2020). Schizotypal and obsessive-compulsive traits: Co-occurrence rate and relationship with executive function, emotion experience, and emotion expressivity in college students. Psych. J..

[55] Fuse-Nagase Y, Miura J, Namura I, Sato T, Yasumi K, Marutani T, Sugita Y (2016). Decline in the severity or the incidence of schizophrenia in Japan: A survey of university students. Asian J. Psychiatr..

[56] Oswalt, S. B. *et al.* Trends in college students’ mental health diagnoses and utilization of services, 2009–2015. *J. Am. Coll. Health* **68**(1), 41–51 (2020). 10.1080/07448481.2018.1515748 30355071

[57] Sun X, So SHW, Chiu CD, Chan RCK, Leung PWL (2018). Paranoia and anxiety: A cluster analysis in a non-clinical sample and the relationship with worry processes. Schizophr. Res..

[58] Harper DJ, Timmons C (2021). How is paranoia experienced in a student population? A qualitative study of students scoring highly on a paranoia measure. Psychol. Psychother. Theory Res. Pract..

[59] Newman-Taylor K, Kemp A, Potter H, Au-Yeung SK (2018). An online investigation of imagery to attenuate paranoia in college students. J. Child Fam. Stud..

[60] Kingston J, Lassman F, Matias C, Ellett L (2019). Mindfulness and paranoia: A cross-sectional, longitudinal and experimental analysis. Mindfulness.

[61] Li, Y., Cao, F., Cao, D. & Liu, J. Nursing students’ post-traumatic growth, emotional intelligence and psychological resilience. *J. Psychiatr. Ment. Health Nurs.* **22**(5), 326–332 (2015). 10.1111/jpm.12192 25524781

[62] Arpawong TE, Sussman S, Milam JE, Unger JB, Land H, Sun P, Rohrbach LA (2015). Post-traumatic growth, stressful life events, and relationships with substance use behaviors among alternative high school students: A prospective study. Psychol. Health.

[63] Perfect MM, Turley MR, Carlson JS, Yohanna J, Saint Gilles MP (2016). School-related outcomes of traumatic event exposure and traumatic stress symptoms in students: A systematic review of research from 1990 to 2015. Sch. Ment. Health.

[64] Thompson, E. C., Andorko, N. D., Rakhshan Rouhakhtar, P., Millman, Z. B., Sagun, K., Han, S., Chibani, D., Reeves, G. M., Herman, B. & Schiffman, J. Psychosis-spectrum screening and assessment within a college counseling center: A pilot study exploring feasibility and clinical need. *J. Coll. Stud. Psychother.*, 1–22 (2020) 10.1080/87568225.2020.1797604 PMC9175646 35694629

[65] Richardson, T., Yeebo, M., Jansen, M., Elliott, P. & Roberts, R. Financial difficulties and psychosis risk in British undergraduate students: A longitudinal analysis. *J. Public Ment. Health* (2018) 10.1007/s10597-016-0052-0 PMC5337246 27473685

[66] Andorko ND, Mittal V, Thompson E, Denenny D, Epstein G, Demro C, Wilson C, Sun S, Klingaman EA, DeVylder J, Oh H (2017). The association between sleep dysfunction and psychosis-like experiences among college students. Psychiatry Res..

[67] Sanderson VA, Vandyk AD, Graham ID, Lightfoot S, Murawsky M, Sikora L, Jacob JD (2020). Post-secondary students with symptoms of psychosis: A mixed-methods systematic review. Int. J. Ment. Health Nurs..

[68] Shi J, Wang L, Yao Y, Chen F, Su N, Zhao X, Zhan C (2016). Protective factors in Chinese university students at clinical high risk for psychosis. Psychiatry Res..

[69] Luo X, Zhang L, Zhang J, Chen H, Hong H, Luo R, Ma L, Wang C, Jin F, Wang E, Jiang Z (2021). Changes in the cognitive function of Chinese college students with a clinical high risk of psychosis. Psychiatry Res..

[70] Wimberly, C. E., Rajapakse, H., Park, L. P., Price, A., Proeschold-Bell, R. J. & Østbye, T. Mental well-being in Sri Lankan medical students: A cross-sectional study. *Psychol. Health Med.*, 1–14 (2020) 10.1080/13548506.2020.1858488 33356528

[71] Rathnayake S, Ekanayaka J (2016). Depression, anxiety, and stress among undergraduate nursing students in a public university in Sri Lanka. Int. J. Caring Sci..

[72] Wickramasinghe ND, Dissanayake DS, Abeywardena GS (2018). Prevalence and correlates of burnout among collegiate cycle students in Sri Lanka: A school-based cross-sectional study. Child Adolesc. Psychiatry Ment. Health.

[73] Madhusanka, A. K. P. *et al.**Factors affecting the level of stress among undergraduates in Sri Lanka with special reference to covid-19* (University of Moratuwa, Digital Library, 2021).

[74] Mahees, M. T. Stress among university undergraduates: A case study of University of Colombo, Sri Lanka. *Int. Educ. Appl. Res. J. (IEARJ)*. **4**(05) (2020).

[75] Kessler RC, Barker PR, Colpe LJ, Epstein JF, Gfroerer JC, Hiripi E, Howes MJ, Normand SLT, Manderscheid RW, Walters EE, Zaslavsky AM (2003). Screening for serious mental illness in the general population. Arch. Gen. Psychiatry.

[76] Lamers SM, Westerhof GJ, Bohlmeijer ET, ten Klooster PM, Keyes CL (2011). Evaluating the psychometric properties of the mental health continuum-short form (MHC-SF). J. Clin. Psychol..

[77] Baker, A., Simon, N., Keshaviah, A., Farabaugh, A., Deckersbach, T., Worthington, J. J., Hoge, E., Fava, M. & Pollack, M. P. Anxiety Symptoms Questionnaire (ASQ): Development and validation. *Gen. Psychiatry*. **32**(6), (2019) 10.1136/gpsych-2019-100144 PMC6936972 31922090

[78] Lasa L, Ayuso-Mateos JL, Vázquez-Barquero JL, Dıez-Manrique FJ, Dowrick CF (2000). The use of the Beck Depression Inventory to screen for depression in the general population: A preliminary analysis. J. Affect. Disord..

[79] Hirschfeld RM, Williams JB, Spitzer RL, Calabrese JR, Flynn L, Keck PE, Lewis L, McElroy SL, Post RM, Rapport DJ, Russell JM (2000). Development and validation of a screening instrument for bipolar spectrum disorder: The Mood Disorder Questionnaire. Am. J. Psychiatry.

[80] Nijenhuis ER, Spinhoven P, Van Dyck R, Van der Hart O, Vanderlinden J (1997). The development of the somatoform dissociation questionnaire (SDQ-5) as a screening instrument for dissociative disorders. Acta Psychiatr. Scand..

[81] Gideon N, Hawkes N, Mond J, Saunders R, Tchanturia K, Serpell L (2016). Development and psychometric validation of the EDE-QS, a 12 item short form of the Eating Disorder Examination Questionnaire (EDE-Q). PLoS ONE.

[82] Mallet J, Lancrenon S, Llorca PM, Lançon C, Baylé FJ, Gorwood P (2018). Validation of a four items version of the Functional Remission of General Schizophrenia scale (the mini-FROGS) to capture the functional benefits of clinical remission. Eur. Psychiatry.

[83] Freeman D, Bird JC, Loe BS, Kingdon D, Startup H, Clark DM, Ehlers A, Černis E, Wingham G, Evans N, Lister R (2020). The Dunn worry questionnaire and the paranoia worries questionnaire: New assessments of worry. Psychol. Med..

[84] Loewy RL, Bearden CE, Johnson JK, Raine A, Cannon TD (2005). The prodromal questionnaire (PQ): Preliminary validation of a self-report screening measure for prodromal and psychotic syndromes. Schizophr. Res..

